# Discovery and Characterization of Small Molecule Inhibitors Targeting Exonuclease 1 for Homologous Recombination-Deficient Cancer Therapy

**DOI:** 10.1021/acschembio.5c00117

**Published:** 2025-05-16

**Authors:** Yixing Wang, Jessica D. Hess, Chen Wang, Lingzi Ma, Megan Luo, Jennifer Jossart, John J. Perry, David Kwon, Zhe Wang, Xinyu Pei, Changxian Shen, Yingying Wang, Mian Zhou, Holly Yin, David Horne, André Nussenzweig, Li Zheng, Binghui Shen

**Affiliations:** 1 Department of Cancer Genetics and Epigenetics, 20220 Beckman Research Institute, City of Hope, Duarte, California 91010, United States; 2 Department of Molecular Diagnostics and Experimental Therapeutics, 20220 Beckman Research Institute, City of Hope, Duarte, California 91010, United States; 3 Department of Molecular Medicine, 20220 Beckman Research Institute, City of Hope, Duarte, California 91010, United States; 4 Laboratory of Genome Integrity, 272101 National Cancer Institute NIH, Bethesda, Maryland 20892, United States

## Abstract

Human exonuclease 1 (EXO1), a member of the structure-specific nuclease family, plays a critical role in maintaining genome stability by processing DNA double-strand breaks (DSBs), nicks, and replication intermediates during DNA replication and repair. As its exonuclease activity is essential for homologous recombination (HR) and replication fork processing, EXO1 has emerged as a compelling therapeutic target, especially in cancers marked by heightened DNA damage and replication stress. Through high-throughput screening of 45,000 compounds, we identified seven distinct chemical scaffolds that demonstrated effective and selective inhibition of EXO1. Representative compounds from two of the most potent scaffolds, C200 and F684, underwent a comprehensive docking analysis and subsequent site-directed mutagenesis studies to evaluate their binding mechanisms. Biochemical assays further validated their potent and selective inhibition of the EXO1 nuclease activity. Tumor cell profiling experiments revealed that these inhibitors exploit synthetic lethality in BRCA1-deficient cells, emphasizing their specificity and therapeutic potential for targeting genetically HR-deficient (HRD) cancers driven by deleterious mutations in HR genes like BRCA1/2. Mechanistically, EXO1 inhibition suppressed DNA end resection, stimulated the accumulation of DNA double-strand breaks, and triggered S-phase PARylation, effectively disrupting DNA repair pathways that are essential for cancer cell survival. These findings establish EXO1 inhibitors as promising candidates for the treatment of HRD cancers and lay the groundwork for the further optimization and development of these compounds as targeted therapeutics.

## Graphical abstract

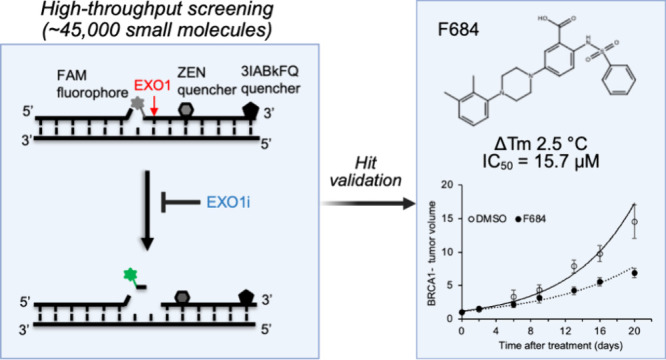

## Introduction

A major disadvantage of conventional chemotherapy is its lack of cancer cell specificity. Therefore, identifying pathways that specifically support cancer cells is key to effective treatment. Cancer cells with activated oncogenes, aberrant checkpoints, and/or aneuploid/polyploid genomes have aberrant DNA replication, which manifests as a high level of double-strand breaks (DSBs) due to stalling and collapse of DNA replication forks.^1–5^ Given that a single unrepaired DSB may cause cell death,^6,7^ most chemotherapies also target replication forks to directly or indirectly induce DNA DSBs.^8^ Targeting both DNA replication and DNA repair has recently been proposed as a major opportunity to enhance current cancer therapies based on DNA-damaging agents by blocking DSB repair.^9,10^ Recent advancements in describing the mechanisms of DNA replication in cancer cells under stress conditions suggest that aberrant DNA replication and repair processes in cancer cells are interconnected.^11^ Thus, we propose that one way to maximally harness DSBs in cancer cells for cancer therapy is to target the replication–repair interface that processes replication intermediates and repairs replication-associated DSBs.

Exonuclease 1 (EXO1) is a key enzyme in the replication–repair interface. EXO1 is an evolutionarily conserved member of the XPG/Rad2 family of nucleases and plays a multifaceted role in DNA metabolic pathways governing genome stability, including double-strand break repair (DSBR), mismatch repair, telomere maintenance, restarting of stalled replication forks, and lagging DNA strand maturation.^12–15^ It is an important nuclease involved in Okazaki fragment maturation (OFM), the process by which RNA–DNA flaps are removed to generate ligatable nicks for the ligation of individual Okazaki fragments into the intact lagging DNA strand during replication.^16–18^ If the OFM machinery is inhibited, the persistent presence of flap and nick structures behind the advancing replication fork makes the cell highly susceptible to apoptosis, as significant DNA replication stress and double-strand breaks (DSBs) will occur. Blocking nucleases that are essential to the OFM process, such as EXO1, would generate persistent flap/nick substrates in the S phase of the cell cycle and subsequently cause DSBs, particularly in cancer cells, which already bear an increased burden of replication stress.^16–19^ Meanwhile, EXO1 also plays a crucial role in repairing DSBs, including those from the collapsed or cleaved replication forks. The primary function of EXO1 is considered to be its role in DSB end resection to generate 3′ ssDNA overhangs,^20^ which in turn activate the master signaling kinase ATR^21–23^ and promote the assembly of a RAD51-loaded nucleoprotein filament that invades the sister chromatid DNA to initiate DSB repair (DSBR) via homologous recombination (HR).^24–26^ As such, DNA end resection is a critical step in DSB repair by HR. Given that cancer cells have an increased reliance on EXO1 to counteract stress due to abnormal and overactive DNA replication processes, small molecules targeting EXO1 represent a major opportunity to specifically kill cancer cells by targeting aberrant replication-associated repair systems, particularly in human breast cancer and other hormone-dependent cancers that bear an increased burden of replication stress due to heightened hormone signaling.^27,28^

Given its dual role in DNA replication and DSB repair, EXO1 not only is an attractive target for cancer cell-specific killing but also has high potential for inducing synthetic lethality (SL) of cancer cells. Accordingly, we reasoned that a small molecule EXO1 inhibitor (EXO1i) would not only impair OFM, causing persistent single-strand DNA (ssDNA) breaks with the potential for conversion into DSBs, but also simultaneously block DSB repair by blocking the end resection required for HR. This strategy has potential to induce SL in human cancers with pre-existing HR gene deficiencies (HRD), e.g., BRCA1/2 mutations, as well as sensitize cancer cells to PARP inhibitors (PARPis), which are the current standard treatment for HRD cancers.^29–31^ In addition, we posit that EXO1i(s) would display greater specificity than PARPis for SL with HRD because PARPs participate in a wide array of other cellular processes such as transcription and translation, whereas EXO1 does not. By itself, EXO1 is also a crucial enzyme for HR; thus, its deficiency would also yield HRD, offering an additional opportunity for SL with PARPi.^32^ Thus, EXO1i-induced HRD may extend PARPi therapy to larger patient populations without HDR gene mutations.

Previously, we have published one small molecule inhibitor of EXO1, C73, identified via virtual screening, although this compound was not a candidate for further development due to its high molecular weight, high total polar surface area, and poor solubility, properties that are considered to be predictors of reduced bioavailability and drug-likeness.^33^ Thus, in the present study, we set out to develop the first selective and effective EXO1i that could be used as both a research tool (i.e., chemical probe) and a preclinical starting point toward the development of a novel therapeutic drug that induces cancer-cell-specific killing via SL. To accomplish this, we purified and untagged full-length human EXO1 protein at large scale for use in a 45,000 small molecule high-throughput screen to identify novel EXO1-selective inhibitors. Compounds were screened via a robust FRET-based exonuclease activity assay developed and validated by our lab. Results of this screen resulted in seven distinct chemical scaffolds with EXO1-selective inhibitory activity that synergized with the BRCA1-deficiency in human cancer cells. Herein, we report on the discovery and mode of action of these novel EXO1-targeted small molecule inhibitors.

## Results

### EXO1 Gene Expression Is Elevated in Multiple Cancers

To determine whether EXO1 is overexpressed in human cancers, we surveyed the TCGA database for EXO1 expression levels. We found that EXO1 gene expression was elevated in multiple types of cancer, including hormone-related breast, ovarian, and prostate cancers (Figure 1a). We then calculated the correlation coefficient to assess the linear relationship between EXO1 and all other genes in the data set. The volcano map depicts the differential correlation coefficients for the Fanconi anemia (FA) pathway and cell-cycle-related genes (Figure 1b). The FA pathway is a replication-coupled DNA repair mechanism that is known to enlist several key proteins involved in homologous recombination.^34^ Given that, under normal physiology, EXO1 activity is restricted to DNA replication and is known to functionally intersect with FA and cell cycle genes, it was unsurprising to see that these pathways were the most highly correlated with EXO1 expression. KEGG pathway enrichment analysis also confirmed a strong codependency between EXO1 and Fanconi-anemia-related genes and a negative correlation with cell-cycle-related genes (Figure 1c). These findings are consistent with an important role of EXO1 in DNA replication and DNA damage repair, which are aberrantly high in cancer cells relative to their normal tissue counterparts, and suggest that EXO1 may be a promising novel target for anticancer treatment.

**Figure 1:**
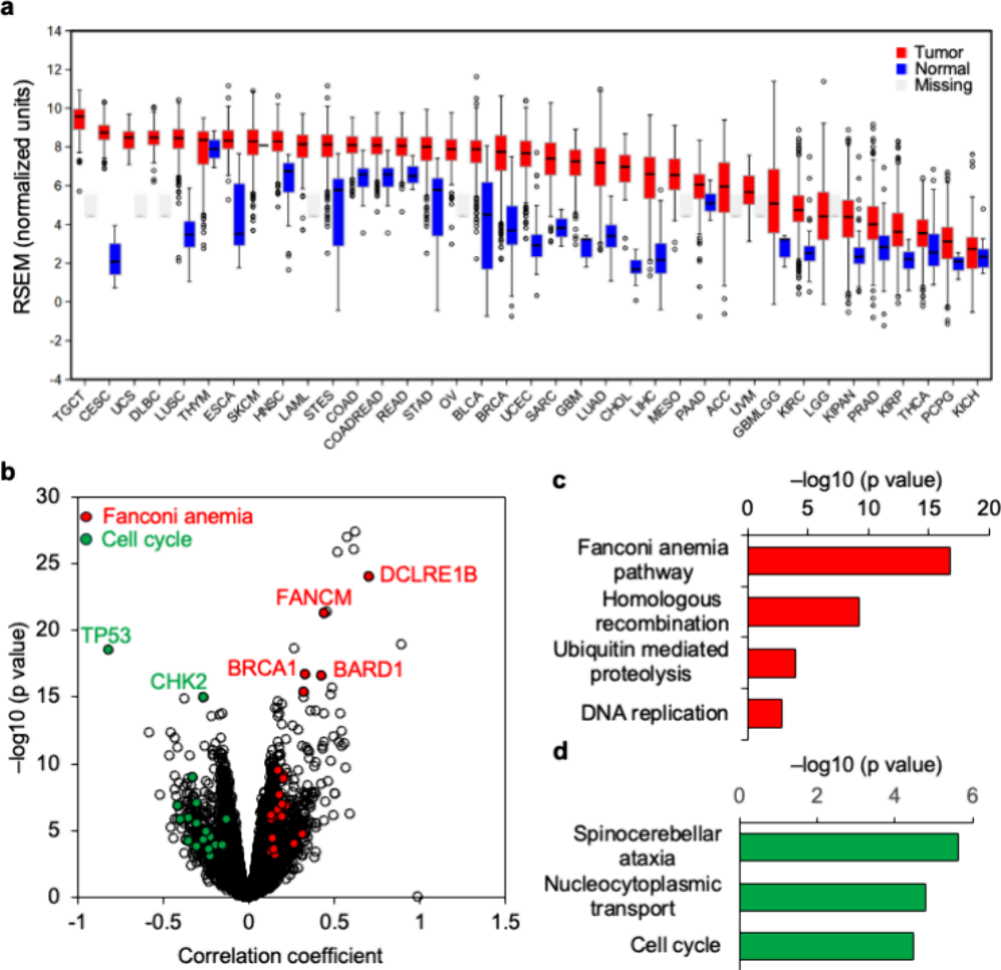
EXO1 expression and gene correlations in cancer. (a) Comparison of EXO1 mRNA expression profiles in cancer and corresponding normal human tissues from the TCGA database. Red bars represent EXO1 expression in cancer tissues; blue bars represent EXO1 expression in corresponding normal human tissues. (b) Volcano map depicting the correlation coefficients for the linear relationship between EXO1, Fanconi anemia, and cell cycle pathway gene expression. Red: Fanconi-anemia-related genes. Green: cell-cycle-related genes. (c, d) KEGG pathway enrichment analysis highlighting gene pathways that are (c) codependent on EXO1 and (d) negatively correlated with EXO1 expression.

### High-Throughput Screening Reveals Seven Distinct Inhibitor Scaffolds

Traditionally, our group has used the gold-standard ^32^P-labeled substrate-based *in vitro* nuclease assay to characterize the nuclease activities of EXO1 and other structure-specific nucleases, including FEN1 and DNA2.^35^ Although this conventional assay provides high resolution of DNA cleavage by nucleases, it is unsuitable for high-throughput screening (HTS) for inhibitors due to its low throughput and use of radioisotopes. To overcome these limitations, we developed an *in vitro* assay for EXO1 exonuclease activity based on fluorescence resonance energy transfer (FRET) quenching, which is amenable to HTS for inhibitors (Figure 2a).

**Figure 2:**
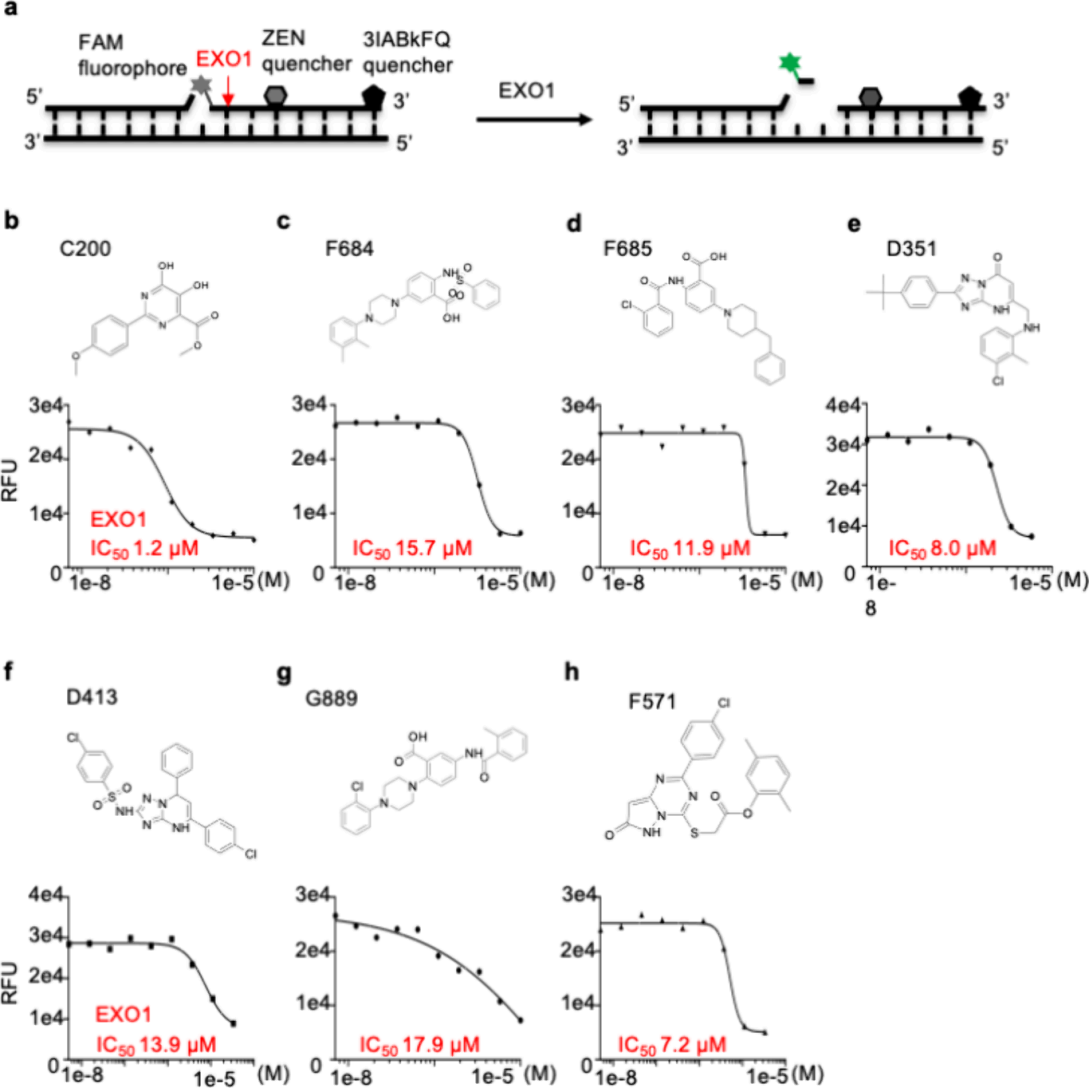
IC_50_ of inhibitor scaffolds against the EXO1 protein measured using a FRET-based high-throughput screening assay. (a) Schematic of the assay. The nick substrate is labeled with FAM fluorophore at the 5′ end of the downstream oligo, which also has an internal ZEN quencher and a 3′ IowaBlackFQ 3IABkFQ quencher. Cleavage of the nick substrate by EXO1 lights up the FAM to give green fluorescence. (b–h) Dose–response curves and calculated IC_50_ values of the seven inhibitor scaffolds against the EXO1 protein.

To establish optimal HTS parameters, we systemically characterized the kinetics of the EXO1 exonuclease activity using our FRET-based assay. We found that at a concentration of 100 pM EXO1, reactions had a sufficiently high ratio (∼10-fold) of product to background fluorescence, allowing us to detect as little as 10% product, which is ideal for kinetic analysis. Using 100 nM DNA substrate, the linear regions of the progress curves (relative fluorescence unit [RFU] vs time) were 10, 15, 20, 30, and 40 min (Figure S1a). Furthermore, we tested the enzymatic activity of EXO1 (100 pM) in the presence of varying concentrations of the DNA substrate and found that at a 200 nM concentration of the substrate, the fluorescent signal intensity was sufficiently above background (Figure S1b). We further tested if DMSO interfered with fluorescence intensity and observed that DMSO had a minimal impact on EXO1 activity even at a final concentration of 10% (v/v) within 120 min (Figure S1d,e). Finally, we evaluated the stability of purified EXO1 protein in a reaction buffer maintained on ice for 1, 2, 4, 8, 12, or 16 h. The retained activity levels were approximately 100, 100, 100, 98, 85, and 83% of the activity measured at 0 h, respectively (Figure S1c). The EXO1 protein in the stock buffer maintained at RT for 16 h still demonstrated full activity (Figure S 1f). We thus selected 100 pM EXO1 and 200 nM DNA substrate concentrations and up to 30 min as the reaction end point during HTS so that the reaction remained in the linear phase.

Using our FRET-based exonuclease assay, a series of EXO1 inhibitors were identified from HTS of the 45,000-compound Targeted Diversity library from ChemDiv. There were a total of 20 confirmed hits whose inhibitory activity (IC_50_) against EXO1 was 20 μM or less (data not shown). These hits were cheminformatically grouped into seven different chemical scaffolds. The best compound within each chemical scaffold is presented as an example in Figure 2b–h. We further tested these compounds’ specificity by comparing their activities against EXO1 vs FEN1 protein (Figure S2), which is functionally related to and occasionally compensated for during replication by EXO1. In yeast, the nuclease domain of Exo1 and FEN1 (Rad27) share 28% identity,^36^ while in humans, they share 29.8% identity.^37^

Most of the EXO1 inhibitor scaffolds showed no or weak inhibitory effect on FEN1 exonuclease activity (>100 μM; Table 1, Figure S3a–g). Compounds displaying a favored inhibition against EXO1 rather than FEN1 (with an IC_50_ ratio >5) were prioritized. Most confirmed hits passed this criterion, with the exception of two compounds within the F685 scaffold (data not shown). A total of 18 inhibitors showed strong inhibition of EXO1 at low micromolar concentrations (data not shown), with preferred inhibition against EXO1 rather than FEN1, which is suggestive of a potential for further structural optimization.

**Table 1:** Selective Inhibition and Thermal Stabilization of EXO1 by Inhibitor Candidates

compound	EXO1 IC_50_ (μM)	FEN1 IC_50_ (μM)	Δ*T*_m_ (°C)
C200	1.2	>100.0	1.3
F684	15.7	>100.0	2.5
F685	11.9	>100.0	3.0
D351	8.0	>100.0	3.0
D413	13.9	>100.0	3.5
G889	17.9	>100.0	1.5
F571	7.2	>100.0	5.0

### Inhibitors Stably Bind to EXO1

The melt curves and melting temperature (*T*_m_) of the protein in the presence of increasing concentrations of inhibitors are depicted in Figure 3a,b and Figure S4. The panels illustrate the derivative of the thermal shift assay melt curves and display the maximum shift in *T*_m_ for each compound. Inhibitors C200 and G889 exhibited a minimal change (∼1 °C) in *T*_m_ across the tested concentrations, up to 100 μM, suggesting that they may weakly bind to EXO1 (Figure 3a, Figure S4b). Of note, the molecular weight of C200 classifies it as a “fragment” hit (<300 Da). Due to their smaller size and generally weaker binding affinity, fragments typically exhibit lower *T*_m_ shifts compared to larger molecules.^38^ In contrast, the binding curves for inhibitors F684, D351, D413, F571, and F685 demonstrate a clear ≥2 °C increase in *T*_m_ with higher inhibitor concentrations, indicating a significant stabilizing effect that is indicative of strong binding to the EXO1 protein (Figure 3b, Figure S4a,c–e). Overall, these results provide valuable insights into the strength of binding for each inhibitor at the EXO1 nuclease domain, which could be critical for further drug development and optimization.

**Figure 3:**
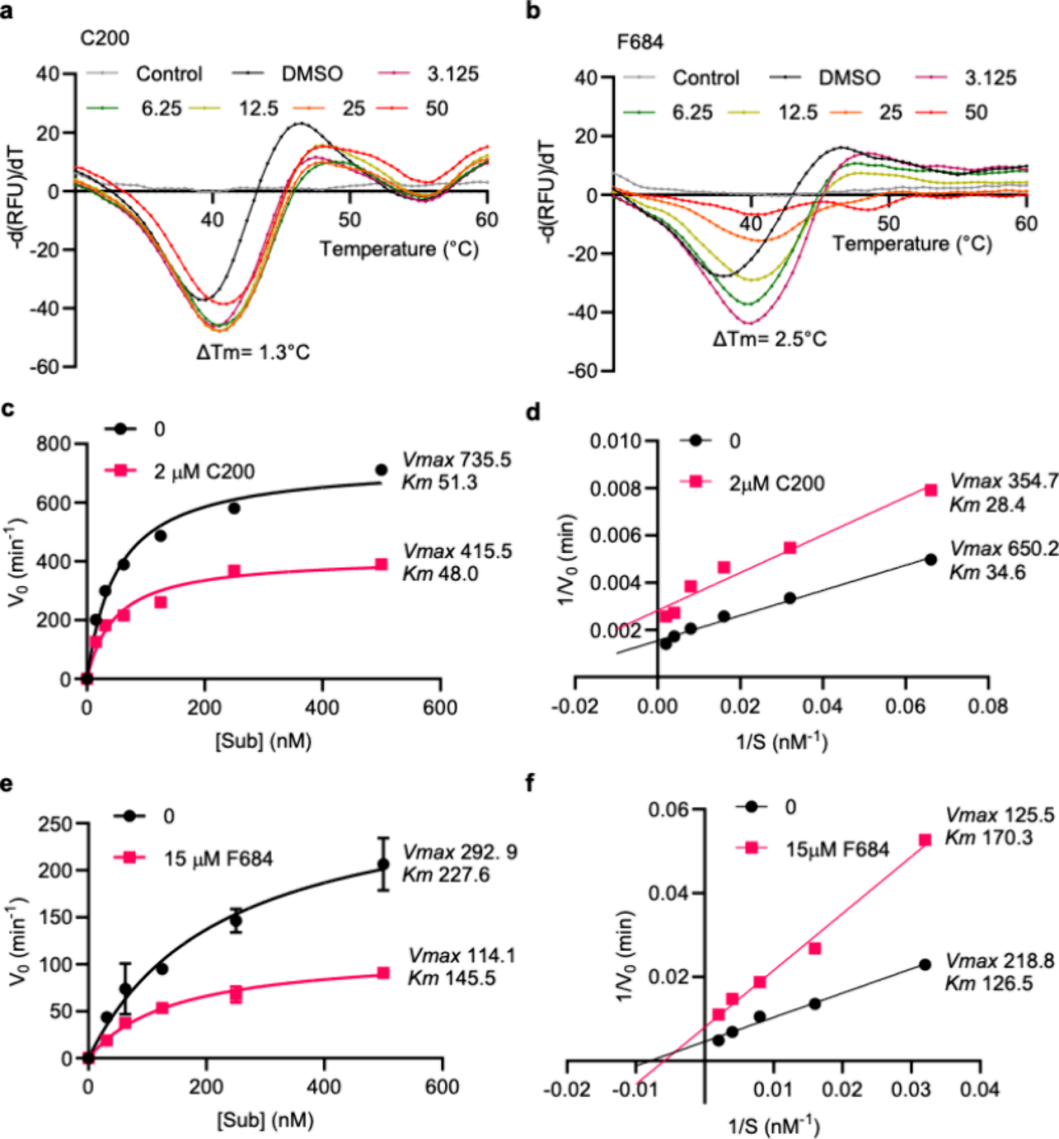
Enzyme inhibition kinetics of compounds C200 and F684. Derivative of thermal shift assay melt curves for compound C200 (a) and F684 (b) with their corresponding maximum Δ*T*_m_. Michaelis–Menten plots: (c) DMSO exhibits higher *V*_max_ (735.5 min^–1^) and *K*_m_ (51.3 nM) values compared to C200 (*V*_max_: 415.5 min^–1^, *K*_m_: 48.0 nM), indicating reduced catalytic efficiency with C200 treatment. (e) F684 significantly lowers *V*_max_ (114.1 min^–1^) compared to DMSO (292.9 min^–1^), while *K*_m_ decreases (145.5 vs 227.6 nM), indicating strong inhibition. Lineweaver–Burk plots: (d) Linearized data show *V*_max_ and *K*_m_ values for DMSO (650.2 min^–1^, 34.6 nM) and C200 (354.7 min^–1^, 28.4 nM), supporting a decrease in enzyme activity with C200. (f) *V*_max_ and *K*_m_ values for DMSO (218.8 min^–1^ and 126.5 nM, respectively) and F684 (125.5 min^–1^ and 170.3 nM, respectively) reveal reduced enzymatic efficiency and substrate affinity in F684-treated samples.

### Prioritization of EXO1 Inhibitor Candidates for Further Analysis

We next evaluated the physicochemical properties of each inhibitor candidate via the SwissADME web tool to estimate their pharmacokinetics and drug-likeness to determine which compounds to prioritize for further analysis. For comparison, the previously published EXO1 inhibitor C73 was also included in the analysis. Six key parameters were considered: lipophilicity (LIPO), size (MW), polarity (POLAR), water solubility (INSOLU), saturation (INSATU), and flexibility (FLEX). Compounds that fall within the optimal ranges for each of these six parameters have been correlated with favorable absorption, distribution, metabolism, and excretion (ADME) profiles. The optimal ranges for each property were considered as follows: LIPO (using XLOGP3) between −0.7 and +5.0; MW between 150 and 500 g/mol; POLAR (TPSA) between 20 and 130 Å^2^; INSOLU (using Log *S* (ESOL)) between −6 and 0; INSATU (Fsp3) between 0.25 and 1; and FLEX (number of rotatable bonds) between 0 and 9.

As detailed in Table 2, SwissADME analysis revealed that the majority of compounds fell within the optimal range for five of the six parameters: lipophilicity, size, polarity, water solubility, and flexibility. The only consistently out-of-range property was saturation, which fell below the recommended threshold of 0.25 for all but one compound.

**Table 2:** Physicochemical Properties of Novel EXO1 Inhibitor Candidates and C73

compound	LIPO	MW	POLAR	INSOLU	INSATU	FLEX
C200	3.21	274.27	75.99	–3.74	–3.74	4
F684	4.97	465.56	98.33	–5.87	–5.87	6
F685	6.29	448.94	69.64	–6.54	–6.54	7
D351	6.31	436.96	77.88	–6.48	–6.48	5
D413	4.99	513.42	94.54	–6.23	–6.23	5
G889	4.99	449.93	72.88	–5.79	–5.79	6
F571	3.66	455.94	117.45	–4.86	–4.86	6
C73	4.14	546.6	164.26	–5.45	–5.45	11

C200 had the most promising overall profile, displaying the lowest molecular weight (274.27 g/mol), moderate lipophilicity (XLOGP3 = 3.21), moderate polarity (TPSA = 75.99 Å^2^), high water solubility (ESOL = −3.74), and moderate flexibility (rotatable bonds = 4). Similarly, F684 fell within the acceptable range for size (MW = 465.56 g/mol), lipophilicity (XLOGP3 = 4.97), polarity (TPSA = 98.33 Å^2^), water solubility (ESOL = −5.87), and flexibility (rotatable bonds = 6) and outperformed all but one compound (i.e., D351) in terms of saturation (Fsp3 = 0.24). In contrast, the remaining five scaffolds displayed multiple red flags: F685 and D351 are excessively lipophilic, D413 and C73 have high molecular weights, and F571 and C73 are highly polar. As expected, C73 did not perform well in this analysis and fell within the optimal range for just two of the six properties (i.e., lipophilicity and water solubility). Accordingly, C200 and F684 were prioritized for further analysis based on their balanced physicochemical profiles and alignment with drug-likeness criteria.

### Inhibitors Block EXO1 Nuclease Activity via an Uncompetitive Mechanism

To determine the IC_50_ of our EXO1i using a flap DNA substrate that we intended to use for the study of their binding mechanism, we conducted inhibitor kinetic assays. First, we studied the time course of the nuclease activity by comparing the reaction rate with and without inhibitor treatment to determine the 50% inhibitory concentration for kinetic analysis at a 100 nM DNA substrate concentration (Figure S5a). Next, to evaluate the optimal DNA substrate concentrations for enzyme kinetic assays, we measured the relative activity of EXO1 across varying concentrations of substrate and inhibitor and found that the activity was highly consistent across substrate concentrations ranging from 62.5 to 500 nM using inhibitor concentrations up to 10 μM (Figure S5b).

Subsequently, we set out to characterize the inhibitory binding mechanism of C200 and F684 via Michaelis–Menten modeling. For this experiment, we performed the FRET-based EXO1 activity assay at a fixed inhibitor concentration, which corresponded to the IC_50_ of each compound (2 μM for C200 and 15 μM for F684), with varying substrate concentrations: 500, 250, 125, 62.5, 31.25, and 15.625 nM (Figure 3c,e). We then calculated the initial velocity of enzyme activity by calculating the rate within the linear region of the time-dependent RFU. In the absence of an inhibitor, we observed that *V*_max_ = 735.5 min^–1^ and *K*_m_ = 51.3 nM. At 2 μM C200, *V*_max_ was significantly decreased to 415.5 min^–1^, while *K*_m_ showed a modest reduction to 48 nM, suggesting an uncompetitive inhibition mechanism (Figure 3c). We also observed a similar trend in *V*_max_ and *K*_m_ reduction in an independent experiment with F684. *V*_max_ was reduced from 292.9 to 114.1 min^–1^ upon the addition of F684, while *K*_m_ decreased from 227.6 to 145.5 nM (Figure 3e), which is consistent with the improved binding and inhibitory capacity of F684 compared to C200 in our molecular docking, thermal shift, and cell-based assays. Of note, although the baseline *V*_max_ and *K*_m_ values differed significantly between these two experiments, the effect of both compounds on *V*_max_ and *K*_m_ was consistent; thus, we attributed these differences to routine variances in purity and enzyme activity that can occur between batches. To confirm these changes in *V*_max_ and *K*_m_, we constructed a Lineweaver–Burk plot by taking the double reciprocal of the Michaelis–Menten analysis and obtained a similar result (Figure 3d,f).

Based on our findings from both the Michaelis–Menten and the Lineweaver–Burk plots, we concluded that C200 and F684 inhibit EXO1 activity through an uncompetitive mechanism. This suggests that both inhibitors interact with the EXO1/substrate complex in a region near the active site. Upon binding, C200 and F684 can potentially trap EXO1 on the substrate or sterically block scissile bond alignment in the active site, consequently preventing catalysis from occurring. As such, while the affinity of EXO1 for the DNA substrate is maintained, the portion of enzymes that are inhibitor-bound is “catalytically dead” or unable to cleave DNA.

### Identification of Protein Residues That Directly Bind to the Inhibitor

We utilized a 3D structural model for virtual screening of small molecules that bind to EXO1. We predicted druggable sites on the EXO1 model using an in-house-developed Druggable Site Prediction by FDA-approved drugs (DSP) methodology, which uses a diverse subset of 100 FDA-approved drug molecules to dock around the protein surface and predict the best inhibitor binding sites on the protein surface. The “best binding site” was defined as the protein pocket at which the highest number of FDA-approved drugs was bound. We identified two potential inhibitor binding pockets for screening, designated as sites 1 and 2. Site 1 is a well-defined pocket that is in close proximity to the DNA substrate, and site 2 is a shallow surface pocket at an allosteric site within the proximal end of the EXO1 C-terminal domain in an area of unknown function (Figure S6a,b).

To validate the binding site of our inhibitor candidates, we expressed and purified recombinant EXO1 protein with point mutations to residues K85, E89, R92, I125, or D225 in the site 1 pocket or dual mutation to K292 and K294 on the site 2 surface (Figure S7). Each amino acid was mutated to alanine to minimize the potential of side chain interactions or interference. The amino acids were chosen based on a multistructure induced-fit docking analysis to predict the specific residue interactions and potential conformational preference of our seven inhibitor scaffolds with EXO1 throughout its exonucleolytic reaction cycle. The six selected point mutations were chosen due to their frequent direct contact with the inhibitors across the molecular docking models (Figure S8a,b). Residues that are known to be critical for EXO1 nuclease function were excluded from mutagenesis (i.e., Y32 and H36).

To further evaluate the impact of the selected site 1 and site 2 point mutations on inhibitor binding, C200 was docked onto models of the site 1 and site 2 mutants (PDB: 3QEB), and the bound positions and docking scores were compared to the corresponding wild-type protein model (Figures S9 and S11). We found that among the site 1 mutants, R92A and D225A mutants resulted in a significant change in C200 binding position. The detailed position changes are shown in Figure S10a. In contrast, the K85A, E89A, and I125A mutants showed minimal change to the C200 binding position (Figure S10b). As for site 2, the mutation of K292A and K294A resulted in a deviated C200 pose with distance measurements from the wild-type docking position as shown in Figure S12. Individual docking scores for C200 and F684 against each mutant are given in Table S2.

Simultaneously, we tested the effect of each point mutation on the *in vitro* exonuclease activity. Most mutations in site 1 result in some loss of enzymatic activity compared to WT EXO1 (Figure S13; WT:Figure S13a), particularly K85A and D225A (Figure S13b,f). However, E89A and site 2 mutation K292A-K294A showed only minimal loss of enzyme activity (Figure S13c,g). We then tested the activity of each mutant protein with the inhibitors C200 and F684. We found that while C200 effectively blocked the exonuclease activity of the WT EXO1 enzyme, it partially lost its inhibitory effect against the R92A, D225A, and K292A-K294A mutants as the inhibitor concentrations increased (Figure 4a). Compound F684 also partially lost its inhibitory effects against the K85A and R92A mutants at low concentrations. However, at concentrations of 20 and 40 μM, the mutants (K85A and R92A) behaved more like WT (Figure 4b). These data suggest that the inhibitory activity of C200 and F684 is primarily mediated by the interaction with EXO1 residue R92 and other nearby residues in site 1, which are in close proximity to catalytic metal ions within the active site of the protein. It also implies that, at least in the case of C200, the compound may additionally be capable of blocking EXO1 nuclease activity via an allosteric interaction with site 2.

**Figure 4:**
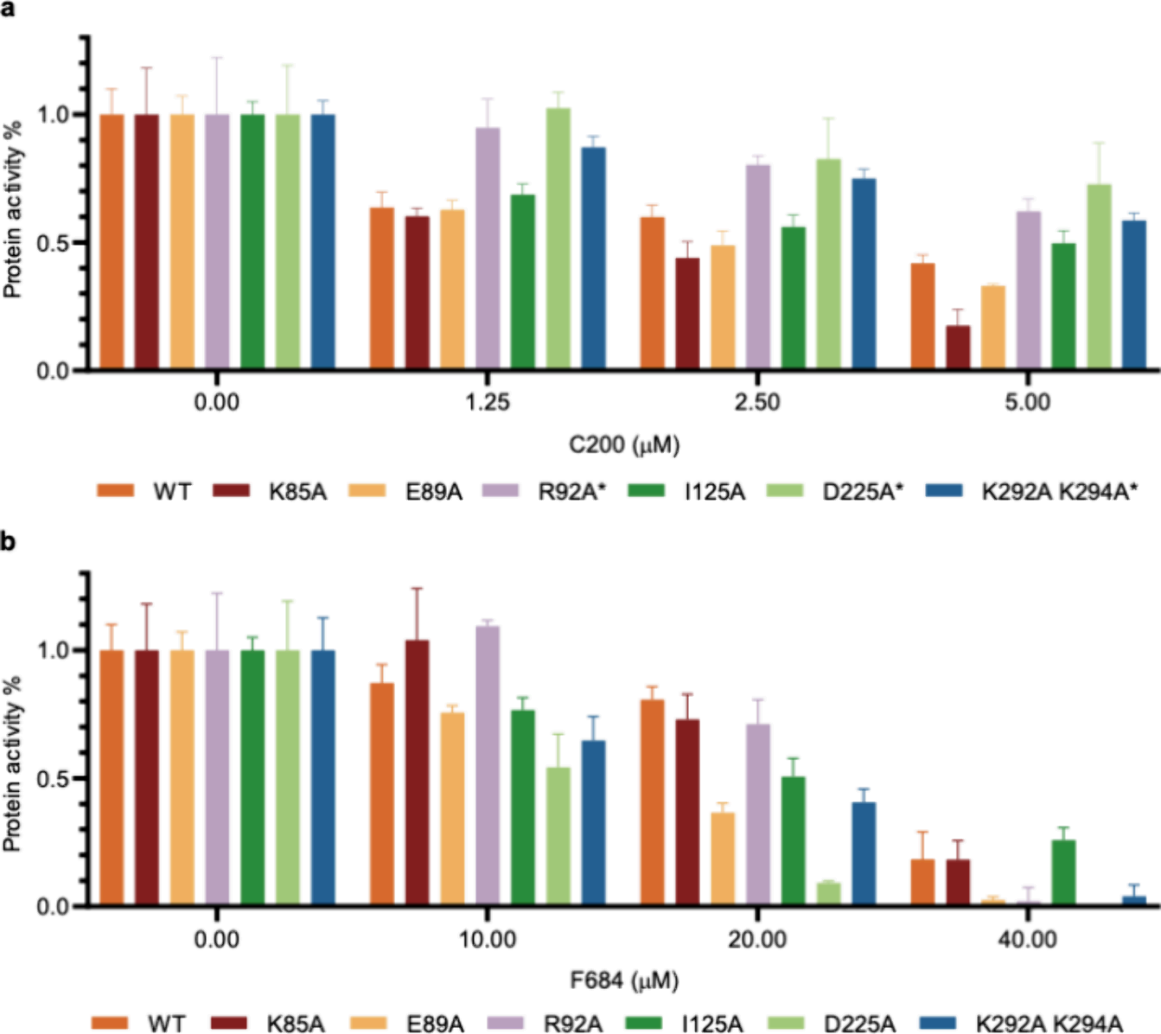
Effect of EXO1 point mutations on C200 and F684 inhibitory activity. (a) The inhibitory activity of compound C200 is abolished in EXO1 site 1 mutants R92A and D225A and site 2 mutant K292A-K294A at C200 concentrations ranging from 0 to 5 μM. (b) The inhibitory activity of compound F684 is abolished in EXO1 site 1 mutants K85A and R92A at 10 μM. F684 concentrations ranged from 10 to 40 μM. In both panels a and b, DMSO without inhibitors was used as a control, and its relative binding activity was set equal to 1.0. The EXO1 enzyme concentration was 25 nM, and the DNA substrate concentration was 250 nM. The values shown are the mean ± S.D. of three independent experiments.

### Treatment with Inhibitors Leads to Accumulation of Double-Strand Breaks and S-Phase PARylation

To study whether unresolved DSBs are involved in the synthetic lethal interaction between BRCA1 deficiency and our EXO1 inhibitors, we analyzed γ-H2AX levels as a marker for unresolved DSBs.^39^ In MDA-MB-436 parental cells, BRCA1-deficiency combined with inhibitor C200 had no effect on γ-H2AX levels (Figure 5a–c), whereas treatment with F684 elicited a significant increase in γ-H2AX after 24 h (Figure 5d–f).

**Figure 5:**
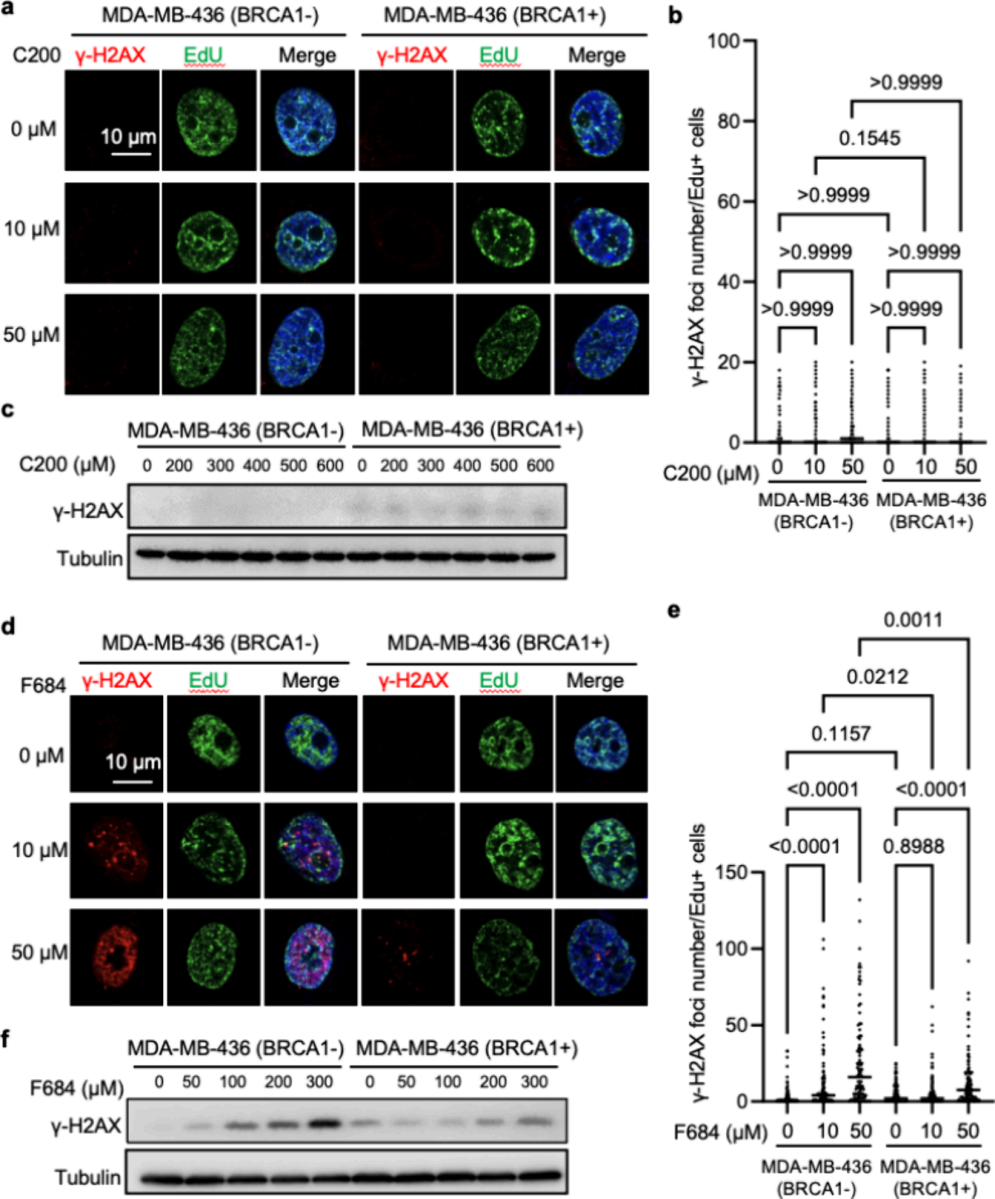
Effects of C200 and F684 on DNA double-strand break burden in MDA-MB-436 breast cancer cells. (a) EXO1 inhibition by C200 did not cause a significant increase in DSB burden. Parental MDA-MB-436 (BRCA1−) and WT BRCA1-expressing MDA-MB-436 (BRCA1+) cells were untreated or treated with 10 or 50 μM C200 overnight. A minimal quantity of γ-H2AX foci was observed; EdU incorporation shows active DNA replication. (b) Quantification of the relative γ-H2AX per nucleus from panel a. (c) Western blot analysis of γ-H2AX after increasing concentrations of C200 treatment. The intensity of the γ-H2AX bands correlates with the level of DSBs observed in the immunofluorescence images in panel a. (d) EXO1 inhibition by F684 significantly increased the level of DNA damage. Parental MDA-MB-436 (BRCA1−) and WT BRCA1-expressing MDA-MB-436 (BRCA1+) cells were untreated or treated with 10 or 50 μM F684 overnight. Strong γ-H2AX foci are observed, indicating an increased burden of DSBs. EdU incorporation shows active DNA replication and partial colocalization with γ-H2AX, suggesting that the DNA damage is replication-associated. (e) Quantification of the relative γ-H2AX per nucleus is depicted in panel d. (f) Western blot analysis of γ-H2AX after increasing concentrations of F684 treatment. The intensity of the γ-H2AX bands correlates with the level of DSBs observed in the immunofluorescence images in panel d.

Additionally, we measured S-phase poly­(ADP-ribosyl)­ation (PARylation), which has been reported to reflect the presence of ssDNA gaps and, more particularly, unprocessed Okazaki fragments, although it is also induced at DSBs and other lesions. Similar to the previous analysis, we observed no effect on S-phase PARylation when EXO1 was inhibited by compound C200 in MDA-MB-436 cells regardless of BRCA1 status (Figure 6a,b), whereas an additive effect on S-phase PARylation was observed with compound F684, particularly in MDA-MB-436 parental (BRCA1-) cells (Figure 6c,d). These results support a potential synthetic lethal relationship between the EXO1 inhibitor F684 and BRCA1 deficiency due to an increased burden of unrepaired DSBs and unligated Okazaki fragments.

**Figure 6:**
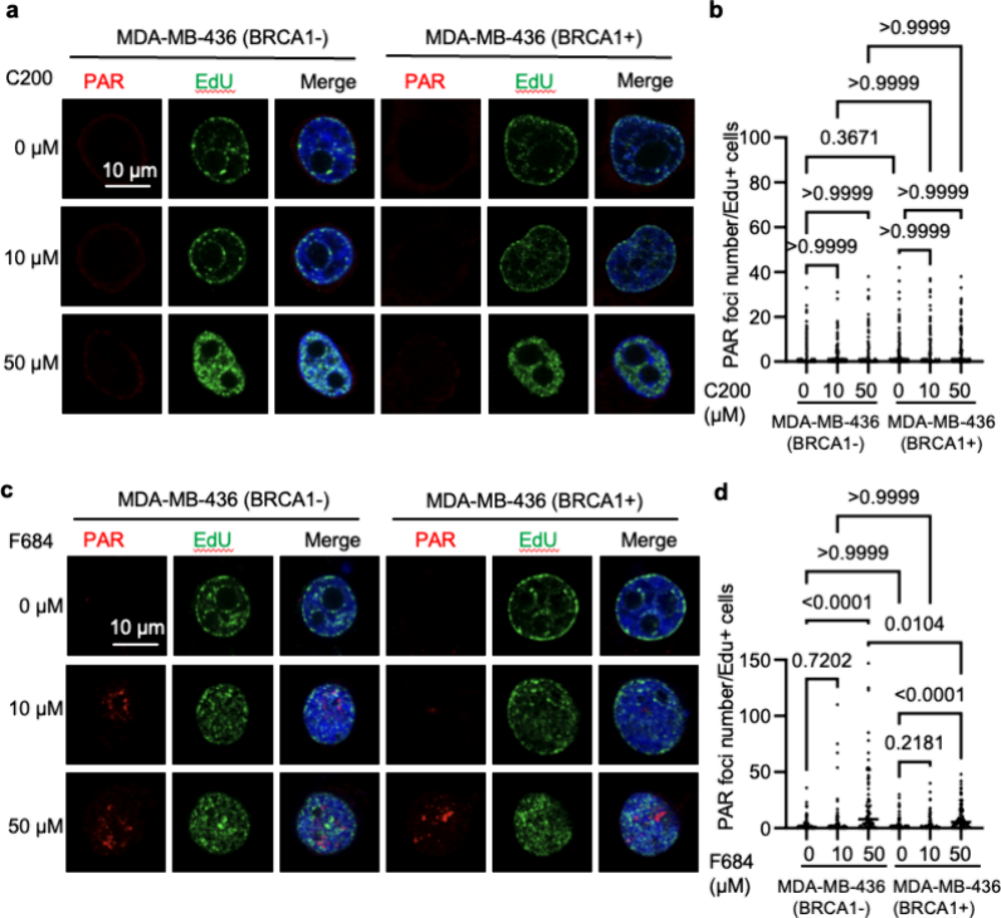
Effects of C200 and F684 on S-phase PARylation in MDA-MB-436 breast cancer cells. (a) EXO1 inhibition by C200 did not significantly increase PARylation. Parental MDA-MB-436 (BRCA1−) and WT BRCA1-expressing MDA-MB-436 (BRCA1+) cells were untreated or treated with 10 or 50 μM C200 overnight. Low PARylation foci levels are observed; EdU incorporation shows active DNA replication. (b) Quantification of the relative PARylation per nucleus from panel a. (c) EXO1 inhibition by F684 significantly increased PARylation. Parental MDA-MB-436 (BRCA1−) and WT BRCA1-expressing MDA-MB-436 (BRCA1+) cells were untreated or treated with 10 or 50 μM F684 overnight. Strong PARylation foci are observed in BRCA1– cells. EdU incorporation shows active DNA replication, and partial colocalization with PARylation suggests that the DNA damage is replication-associated. (d) Quantification of the relative PARylation per nucleus from panel c.

### Inhibitors Block DNA End Resection

We next wanted to verify whether the defects in DSB repair were due to inhibition of end resection. To measure end resection, we determined the level of phosphorylated RPA2 (S33 or S4/8) in MDA-MB-231 cells treated with C200 and F684 in the presence or absence of camptothecin (CPT). CPT stabilizes cleavable complex intermediates in topoisomerase I reactions, which collapse into DSBs when encountered with a replication fork. CPT increased the level of phosphorylated RPA2 (P-RPA) as measured on Western blots. Treatment with F684 or C200 significantly reduced the CPT-induced P-RPA levels, and consistent with our previous findings, DNA2 inhibitor C5 also demonstrated a significant reduction in the CPT-induced P-RPA level that synergized with F684 treatment (Figure 7a,b).^35^

**Figure 7:**
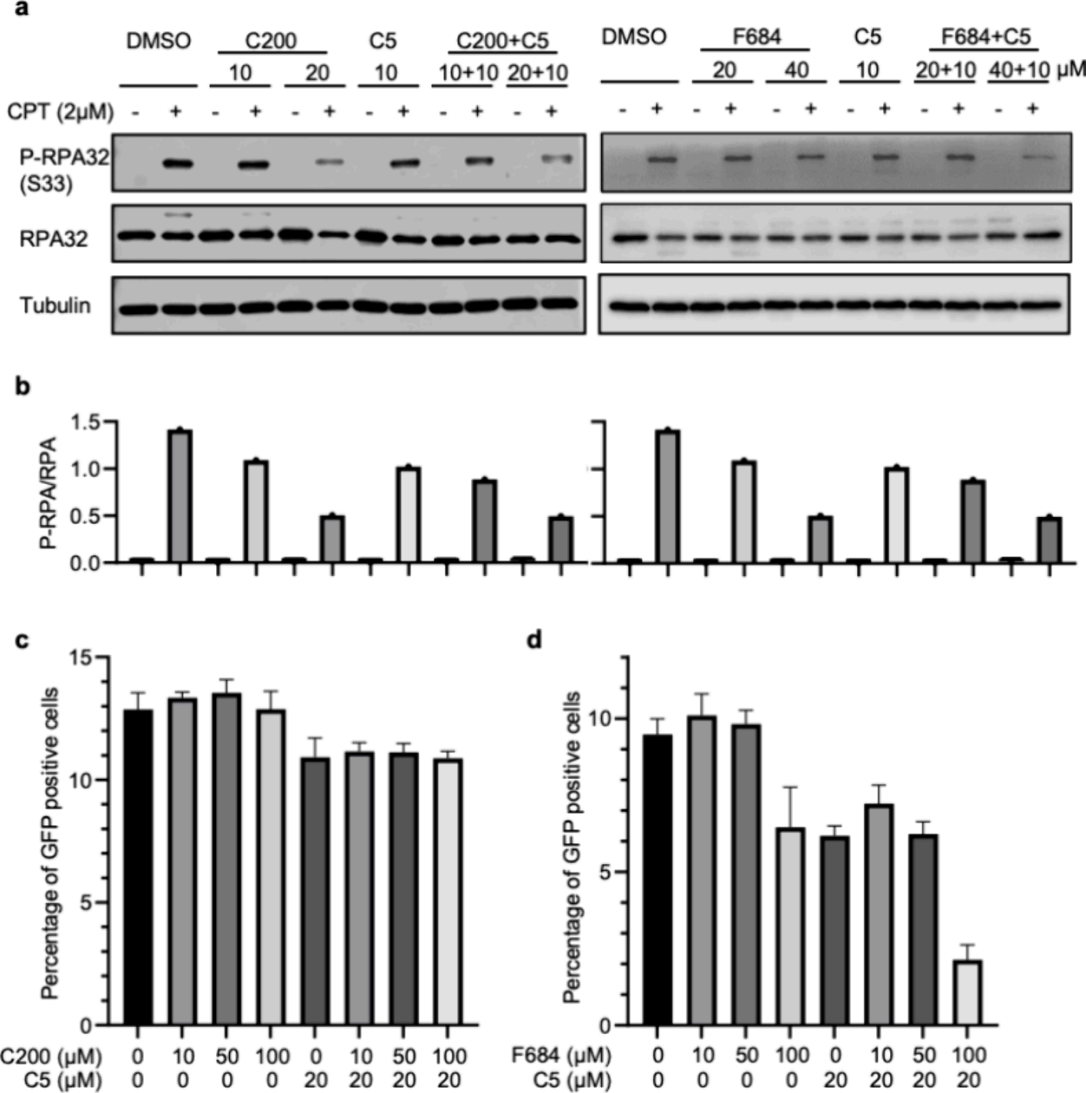
Effects of compounds C200 and F684 on DNA end resection and homologous recombination. (a) EXO1 inhibition by C200 or F684 impaired replication fork-related DNA end resection in MDA-MB-231 breast cancer cells at similar levels. MDA-MB-231 cells were left untreated or treated with 10 or 20 μM C200 overnight (left panels), left untreated or treated with 20 or 40 μM F684 overnight (right panels), or combined with 10 μM C5. The cells were then treated with 2 μM CPT for 2 h. The levels of RPA32 and phosphorylated RPA32 (S33) were analyzed by Western blotting using antibodies against RPA32 and phosphorylated RPA32 (S33). The total level of tubulin was used as a control. (b) Ratio of P-RPA:RPA levels calculated from panel a. (c) C200 does not inhibit homologous recombination frequency. U2OS cells carrying a GFP reporter for HR were infected with adenovirus expressing the I-SceI restriction enzyme. The cells were then incubated in a medium containing 0, 10, 50, and 100 μM C200 or combined with the DNA2 inhibitor C5. (d) F684 inhibits HR frequency. U2OS cells carrying a GFP reporter for HR were infected with adenovirus expressing the I-SceI restriction enzyme. The cells were then incubated in a medium containing 0, 10, 50, and 100 μM F684 or combined with the DNA2 inhibitor C5. For panels c and d, after 48 h, the cells were harvested, and the GFP-positive cells were analyzed by flow cytometry. Values are the mean ± S.D. of three independent experiments.

### Inhibitor F684 Reduces DSB Repair Capacity

To determine whether the reduction in DNA end resection had an effect on the frequency of HR, EXO1i treatment was assessed in U2OS cells harboring GFP-based reporters. Cells were infected with adenovirus expressing the I-SceI restriction enzyme to induce site-specific DNA DSBs and treated with 0, 10, 50, or 100 μM C200 either alone or in combination with the DNA2 inhibitor C5 (10 μM). Consistent with our previous findings, flow cytometry analysis of GFP-positive cells 48 h post-treatment revealed no significant changes in HR frequencies across the C200 treatment groups compared to the untreated control (Figure 7c). These findings suggest that C200, even at higher concentrations, does not impair the repair efficiency of the HR pathway. Next, we treated U2OS cells in an analogous manner, with 0, 10, 50, or 100 μM F684 and with or without C5. In contrast to C200, we found that treatment with F684 resulted in an inhibition of HR that increased with dose, particularly when combined with C5. Flow cytometry showed a marked reduction in GFP-positive cells at 100 μM F684, indicating the significant suppression of the HR pathway, and as expected, the inhibitory effect of F684 was further enhanced when combined with C5 (Figure 7d). These results indicate that F684 binding successfully disrupts the cellular DSB repair by HR.

### Tumor Cell Profiling Reveals Micromolar Cytotoxicity across Multiple Cancer Types and Synergy with BRCA1 Deficiency *In Vitro* and *In Vivo*

We tested a subset of the NCI-60 cell line panel supplemented with an additional three pancreatic cell lines (FG, AsPC1, and CaPan2), totaling 31 cell lines. Inhibitor C200 showed little efficacy in killing cells as the IC_50_ values were greater than 50 μM; F684 was more effective with IC_50_ values generally in the 15–25 μM range (Table S3 and Figure 8a). To further confirm that the cytotoxicity of F684 was selective for BRCA1- cells, Annexin V fluorescence intensity was measured over 5 days of treatment in WT BRCA1-expressing MDA-MB-436 (BRCA1+) and MDA-MB-436 parental (BRCA1−) cell lines (Figure 8b). The conclusions of the Annexin V assay were twofold: (1) F684 treatment in MDA-MB-436 (BRCA1+) cells has a similar effect on cell survival as BRCA1–, and (2) F684 displays a substantially stronger killing effect in a BRCA1– context. Simultaneously, we tested the killing effect of the EXO1 inhibitor F684 *in vitro* and mouse xenograft models using WT BRCA1-expressing MDA-MB-436 (BRCA1+) and MDA-MB-436 parental (BRCA1−) cell lines. *In vitro*, we found that F684 treatment was more effective in killing BRCA1– cells compared to BRCA1+ cells (Figure 8c). Additionally, F684 significantly suppressed growth of BRCA1– tumors but had little effect in suppressing BRCA1+ tumors *in vivo* (Figure 8D,E). At 8.5 mg/kg body weight/day, F684 reduced the average growth rate of BRCA1– tumors from 15.7 to 11.3%/day (*p* = 0.018), while it only reduced the average growth rate of BRCA1+ tumors from 16.6 to 15.1%/day (*p* = 0.64). Together, these findings support the F684 scaffold as an amenable hit for further optimization.

**Figure 8:**
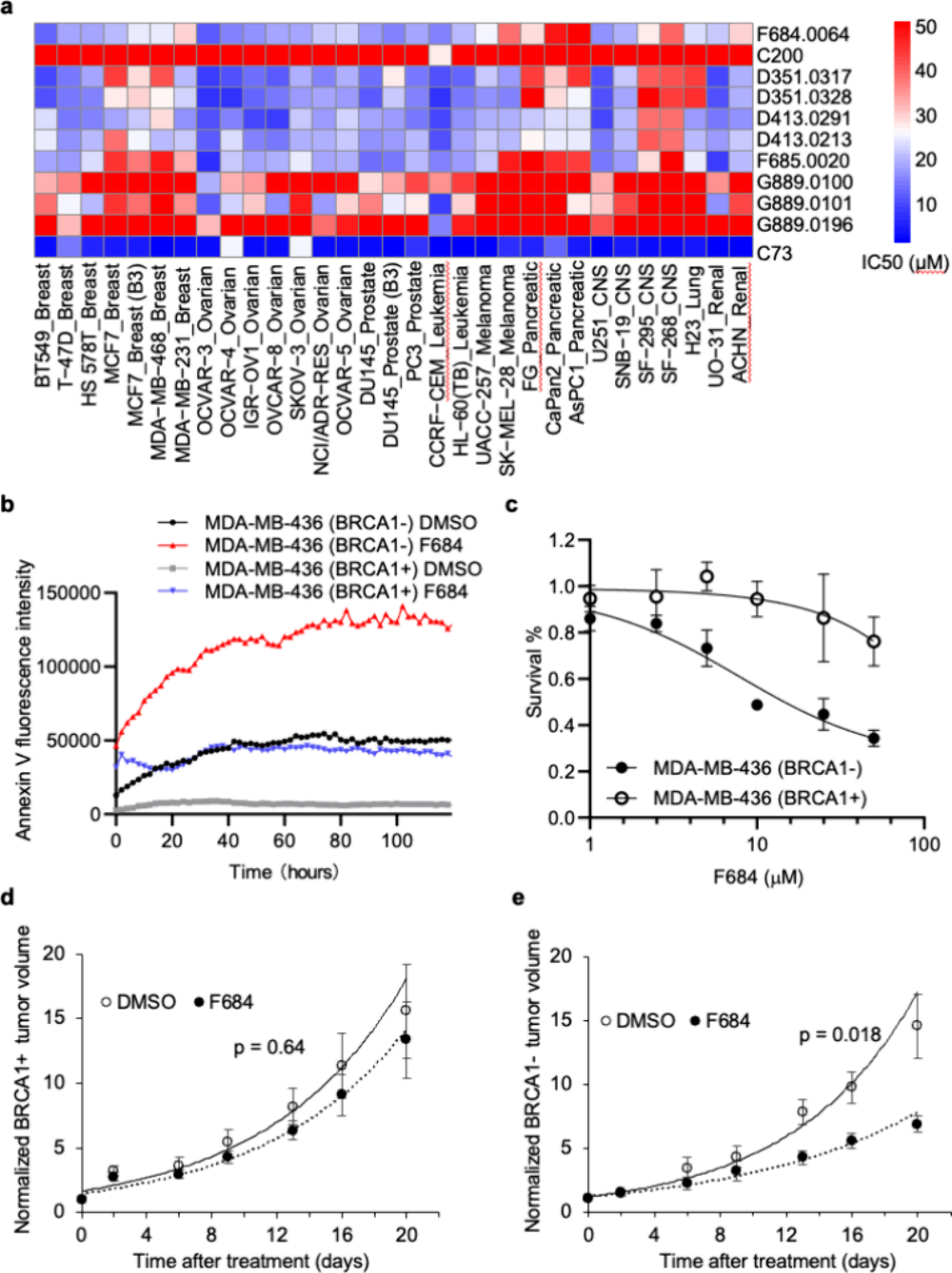
EXO1 inhibitor F684 selectively kills parental MDA-MB-436 (BRCA1−) breast cancer cells both *in vitro* and *in vivo*. (a) IC_50_ values of representative compounds from six of the seven EXO1 inhibitor scaffolds across 31 cancer cell lines compared to the previously reported EXO1 inhibitor C73. Blue color indicates potency <25 μM, white color indicates potency ∼25 μM, and red color indicates potency >30 μM. (b) F684 is substantially more effective at inducing apoptosis in parental MDA-MB-436 (BRCA1−) cells (red line) than WT BRCA1-expressing MDA-MB-436 (BRCA1+) cells (blue line). DMSO controls are shown in black and gray lines, respectively. (c) The cumulative survival percentage of MDA-MB-436 (BRCA1−) and MDA-MB-436 (BRCA1+) cells after treatment with 1, 2.5, 5, 10, 25, and 50 μM concentrations of F684. Error bars represent the standard deviations for each treatment. Similarly, F684 did not reduce the growth rate of BRCA1+ cancer cells in mouse tumor models. Tumor volumes in mouse xenograft models were established using WT BRCA1-expressing MDA-MB-436 (BRCA1+) and parental MDA-MB-436 (BRCA1−) cell lines. Compared with vehicle, F684 showed no effect (*p* = 0.64) in the (d) BRCA1+ tumors but significantly reduced tumor volumes in (e) BRCA1- mouse models (*p* = 0.018). Mean tumor volumes for each group are plotted, and error bars represent the SEM.

## Discussion

Using recombinant full-length human EXO1 protein, we performed a FRET-based high-throughput assay to screen approximately 45,000 NCI library-deposited small molecules to identify EXO1 inhibitors, the hits of which were cheminformatically grouped into seven distinct scaffolds. In this study, we calculated the IC_50_ values of the most potent representative compound from each of the seven inhibitor scaffolds—C200, F684, F685, D351, D413, G889, and F571—using our cell-free FRET-based exonuclease assay system across 31 cancer cell lines of varied origin via CellTiterGlo. Using the most potent, drug-like compounds identified from each of these methods, C200 and F684, respectively, we demonstrate that these compounds inhibit the exonuclease activity of EXO1 biochemically and in breast cancer cells. Additionally, we found that compound F684 effectively reduced the growth of BRCA1– tumors in mice, suggesting that this scaffold is amenable for further optimization.

A wild-type (WT) EXO1 N-terminal domain protein structure (PDB 3QEB; aa 1–352) was used to identify the two binding pockets, designated as sites 1 and 2, where drug-like molecules can preferentially dock. Site 1 is a well-defined pocket located in the active site of the globular N-terminal domain, whereas site 2 is a shallow allosteric surface pocket located on the proximal side of the intrinsically disordered C-terminus. This in-house virtual screening was analogous to the previous method we used to identify DNA2 inhibitors.^35^ Rigid and induced-fit docking methodologies were used to predict the preferred binding site (site 1 vs site 2) and binding poses of these seven inhibitor scaffolds and elucidate the EXO1 residue interactions that drive their inhibitory activity.

The N-terminal domain of EXO1 forms a well-defined bean-shaped core with a helical protrusion and numerous surface grooves. The structure can be divided into four regions, which are defined in reference to the nick at which the enzyme binds: (1) the prenick DNA-binding region, which holds the double-stranded segment with the substrate strand that is cleaved; (2) the postnick binding region, which in the protein binds the single-stranded gap; (3) the active site region, which contains a metal center at which DNA cleavage takes place; and (4) a C-terminal segment.^40^ Our docking model and biochemical data suggest that C200 may bind to both the metal center within the active site region via residues R92 and D225 and the C-terminal segment residues K292 and K294. We propose that this occurs because the molecular weight of C200 (<300 g/mol) is small enough to be considered a “fragment hit”; thus, it may bind to many sites in the EXO1 protein necessary to activate the nuclease. This finding is consistent with the relative inactivity of C200 in our DSB, HR, and cytotoxicity experiments compared to the FRET-based exonuclease assay that was used for HTS. In contrast, while our docking model suggests that F684 may also bind to either site 1 or site 2, at a relatively low concentration of 10 μM, its activity appears to be dependent on its interaction with R92 and K85 within the active site; however, at concentrations above 10 μM, its potential interaction with site 2 may overcome this dependency by inducing a conformational change that directly/indirectly blocks the nucleolytic activity of EXO1. Together, these findings suggest the requirement of both the active site and C-terminal domains for EXO1 nuclease activity. Furthermore, while the active site has been extensively characterized, the distinct mechanisms of these two EXO1 inhibitors may provide a new opportunity to study the helical protrusion and surface grooves, the function of which remain largely conjectural. X-ray crystallography studies are needed to reveal the precise binding relationship between EXO1 and C200 or F684 and provide more definitive insight into the mechanisms we assert for these compounds.

Through a series of functional analyses, we characterized the specific functions of EXO1 that C200 and F684 inhibit. We found that C200 and F684 suppress end resection at CPT-stalled DNA replication forks, as indicated by a decrease in phospho-RPA after treatment, and this effect was more pronounced when treatment was combined with the DNA2 inhibitor C5. Additionally, F684 was capable of selectively inducing replication-coupled DSBs in parental MDA-MB-436 (BRCA1−) cells and attenuating the growth of BRCA1– tumors in a xenograft mouse model. Furthermore, both as a single agent and in combination with C5, F684 caused a significant reduction in the level of homologous recombination. Together, these data support our hypothesis that EXO1 inhibitors can both induce HRD and elicit synthetic lethality in the context of pre-existing genetic defects in HR-related proteins such as BRCA1. In congruence with our biochemical data, F684 also showed much stronger killing effects in cancer cell lines than C200.

The present study adds support to existing evidence that suggests that a major function of EXO1 is to participate in the protection, remodeling, and restart of stalled replication forks. BRCA1 is known to contribute to the expansion of ssDNA gaps, and our group and others have posited that EXO1 becomes essential in BRCA1-deficient contexts. This dependence on DNA end resection may highlight the potential vulnerability of BRCA1 mutant tumors to EXO1 inhibition. Our study additionally supports the notion that EXO1i generate poly­(ADP-ribose)-decorated DNA lesions during the S-phase that are associated with unresolved DSBs and genomic instability in BRCA1-deficient but not in WT BRCA1 cells.^41,42^ PARP inhibitors that were developed for the treatment of cancers with HRD, such as BRCA1– and BRCA2– defective cancers, are a highly successful example of synthetic lethality (SL).^29,43^ Deficiency in any of the homology-directed repair genes has been found to lead to defective DSB repair, thereby promoting PARPi SL. Our finding that EXO1 inhibitors can independently induce HRD implies that they may be effective in both sensitizing cells to PARPi and overcoming resistance to them.

Given that cancer cells require EXO1 to counteract the immense stress of abnormal DNA replication,^44^ small molecules targeting EXO1 exonuclease activity represent a major opportunity for specifically killing cancer cells. Because it is not an essential protein under normal circumstances, EXO1 inhibition would effectively target the defective replication-associated DSB repair systems found in many cancer types, particularly those that are hormone-dependent, already possess mutations in HR proteins like BRCA1/2, or have acquired resistance to other DSB response-targeting agents such as PARPi.

Previously, we had published a small molecule inhibitor of EXO1, C73, identified via virtual screening, whose mechanism has remained largely uncharacterized due to solubility issues.^33^ Discovering and optimizing additional EXO1 inhibitors have great potential for helping to characterize EXO1 enzymatic activities but also offer a new synthetic lethality target for the next generation of anticancer therapies that could be used either as a single agent or in combination with other chemotherapeutics such as CPT or PARPi. Importantly, the chemical inhibition of EXO1 displayed stronger cytotoxicity to BRCA1– cells in our mouse xenograft study. The use and study of EXO1i in cell types that harbor specific mutations in known and unknown EXO1-mediated pathways will help us further define the mechanism of EXO1 nuclease activation and understand its regulation *in vivo*. Small molecule inhibitors, such as those presented in this study, would also facilitate the monitoring of cellular responses to the absence of EXO1, which is an important distinction from genetic studies, whose interpretation is historically difficult for genes with such pleiotropic activity as EXO1.

In this study, we tested if targeting EXO1 may exploit a specific vulnerability in cancer cells, assuming that normal cells are better protected by intact cell cycle checkpoints and redundant DNA repair processes. Our results suggest that EXO1i could be possible therapeutic agents for treating cancers that are defective for HR and other DNA repair and DNA damage checkpoint pathways. Although the cytotoxicity of the present compounds was limited, suitable compounds could be identified using our robust FRET-based exonuclease activity assay and further developed by subjecting them to the other assays introduced in the present EXO1i discovery pipeline. The observation that normal cells can recover from EXO1 inhibition, whereas sensitive cells such as BRCA1-deficient cannot, suggests that cycles of inhibitor treatment might be an effective approach for the clinical use of EXO1i in future studies.

## Materials and Methods

### Differential Expression Analysis

The mRNA expression profiles and correlated clinical data from 33 types of cancer samples and corresponding normal tissue samples were downloaded from TCGA (https://www.cancer.gov/about-nci/organization/ccg/research/structural-genomics/tcga), which involved 11,315 samples in all. The differentially expressed genes (DEGs) between tumors and normal tissues were identified using log2 transformation and *t* tests in the TCGA cohorts with a *p* value < 0.05. The correlation coefficient was calculated from the cancer types that displayed significant differential expression. The Pearson correlation coefficient was computed to assess the linear relationship between EXO1 and all other genes in the data set. The *p* value was calculated using a two-tailed *t* test under the null hypothesis that the correlation coefficient is zero. From this database, we identified the top 100 genes that were most strongly correlated to EXO1 in cancer. Kyoto Encyclopedia of Genomics (KEGG) analyses were subsequently performed for these 100 genes.

### FRET-Based EXO1 Activity Assay and Dose–Response Analysis

We developed a cell-free *in vitro* assay to detect EXO1 exonuclease activity based on FRET quenching. Briefly, a nick substrate was labeled with a FAM (6-carboxyfluorescein) fluorophore at the 5′ end of a downstream oligo. A ZEN quencher and IowaBlackFQ quencher were attached to the downstream oligo internally and at the 3′ end, respectively, to suppress FAM signal. Two quenchers were used to provide better quenching effects than did a single quencher, as recommended by the manufacturer (IDT). The sequence of the nick substrate was as follows: up-primer: GCATCCTAAGCCATGCGTGGC; template: CCTAAAAGTTTCACTCAGGACCACGCATGGCTTAGGATGC; and probe: /56-FAM/TCCTGAGTG/ZEN/AAACTTTTAGG/3IABkFQ/. When EXO1 cleaved the nick substrate, the FAM-labeled nucleotide was untethered from the substrate and yielded a bright green fluorescence. The EXO1 inhibitory capacity of each compound was identified by performing the assay in a 384-well plate format and incubating the FAM-labeled substrate (200 nM) with the reaction buffer only (blank; 50 mM HEPES-KOH (pH 7.5), 45 mM KCl, 5 mM MgCl_2_, 1 mM DTT, 100 μg/mL BSA) or EXO1 (0.25 nM) in the presence of 1% v/v DMSO (vehicle) or candidate EXO1 inhibitor in the reaction buffer at 37 °C. The initial screening of each compound was performed at 10 μM and followed by a dose–response analysis to ascertain the IC_50_ for each compound that displayed significant EXO1 inhibition at the initial concentration. Fluorescent signals were measured after a 30 min incubation. Samples from the initial FRET-based EXO1 activity assay were collected for polyacrylamide gel analysis to confirm that the increases in FAM signal were due to the inhibition of EXO1 activity and not extraneous factors such as inhibitor color interference or quencher technology errors.

Data were normalized by subtracting the relative fluorescence unit (RFU) of the blank sample from that of each EXO1-containing reaction. The relative activity of individual compounds was subsequently calculated by setting the corrected RFU of EXO1 with DMSO as 100% and comparing the corrected RFU for each condition to the DMSO value. Each assay was performed by sequentially adding experimental compounds, the EXO1 enzyme, and the DNA substrate to minimize starting differences. Of the candidate EXO1 inhibitor compounds, hits were chosen if an IC_50_ ≤ 20 μM was observed in the dose–response analysis. Following IC_50_ screening, an analogous FRET-based assay was performed using purified FEN1 to assess the EXO1 specificity of each compound.

### *In Silico* Pharmacokinetic Profiling Using SwissADME

The pharmacokinetic and drug-likeness of individual inhibitor candidates were evaluated using the SwissADME web tool (http://www.swissadme.ch).^45^ The canonical SMILES representations of each compound were manually generated using the Research Collaboratory for Structural Bioinformatics Protein Data Bank (RCSB PDB) Chemical Sketch Tool (https://www.rcsb.org/chemical-sketch) and used as input for the SwissADME interface. Output data were collected for the following physicochemical properties: lipophilicity (Log *P* (using XLOGP3)), size (molecular weight (MW)), polarity (topological polar surface area (TPSA)), water solubility (Log *S* (using ESOL)), saturation (fraction of sp3 carbons (Fsp3)), and flexibility (number of rotatable bonds). Optimal ranges for each of these six parameters were predefined according to SwissADME’s Bioavailability Radar plot, which was developed based on data adapted from the analysis of orally bioavailable drugs.^46–48^ Accordingly, the optimal ranges for each property were considered as follows: Log *P* (XLOGP3) between −0.7 and +5.0; MW between 150 and 500 g/mol; TPSA between 20 and 130 Å^2^; Log *S* (ESOL) between −6 and 0; Fsp3 between 0.25 and 1; and number of rotatable bonds between 0 and 9.

### Virtual Prediction of EXO1 Inhibitor Binding Sites

We predicted druggable sites on the X-ray crystal structure of hEXO1 (PDB: 5V08) using an in-house-developed Druggable Site Prediction by FDA-approved drugs (DSP) methodology, as previously described.^33^ Briefly, a diverse subset of 100 FDA-approved drug molecules was docked around the protein surface to predict the optimal binding site(s) on the protein surface. The most probable inhibitor binding sites were determined based on the locations that contained the highest number of FDA-approved drugs bound to the site.

### Small Molecule Compounds and Human Cell Lines

Each human cancer cell line was cultured according to guidelines from the American Type Culture Collection (ATCC). The corresponding cell culture media were supplemented with 10% fetal bovine serum (FBS) (Cytiva) and an antibiotic solution (Thermo Fisher Scientific) to a final concentration of 100 U/mL penicillin and 100 μg/mL streptomycin. Cells were continuously incubated at 37 °C with 5% CO_2_, except for during passaging, seeding, or physical manipulation of cells for experimental purposes. For high-throughput screening, compounds were acquired from the in-house City of Hope High-Throughput Screening core facility compound library. For subsequent experiments, fresh powders of individual hit compounds were obtained from ChemDiv and camptothecin (CPT) from Selleck Chemicals.

### Thermal Shift Assay

To assess for changes in the thermal stability of EXO1 protein in response to treatment with candidate small molecule inhibitors or DMSO (vehicle control), the thermal shift assay (Applied Biosystems Protein Thermal Shift Dye Kit, Cat. #4461146) was utilized. The protein melt reaction mix consisted of freshly prepared 10× thermal shift dye, purified EXO1 enzyme (1 μg/uL), thermal shift buffer, DMSO or inhibitors, and sterilized Milli-Q water. A drug-free supermix was premade and dispensed in 96-well PCR plates followed by the addition of DMSO or inhibitors with increasing concentration. The reaction plate was heated from 10 to 98 °C at a rate of 0.5° per 10 s cycle, and the fluorescence intensity of individual wells was measured at 490/530 nm using a BioRad CFX96 Touch Real-Time PCR System. The fluorescence measurements of three technical replicates per treatment condition were then averaged to calculate melting temperature shift (Δ*T*_m_) as a readout for compound binding affinity.

### Modeling of Inhibitor Binding Kinetics

To characterize the reaction rate kinetics of F684 and C200 on EXO1 via Michaelis–Menten modeling, we performed the FRET-based EXO1 activity assay at a set inhibitor concentration with varying substrate concentrations. The initial velocity of enzyme activity was obtained by calculating the rate of the linear region of the time-dependent RFU. To observe the change in maximum reaction rate (*V*_max_) and Michaelis constant (*K*_m_), we used the IC_50_ to determine an inhibitor concentration with approximately 50% inhibition and compared it to no inhibitor (0 μM) to confirm our findings. Subsequently, a Lineweaver–Burk plot was generated to confirm the changes in *V*_max_ and *K*_m_.

### Molecular Docking Analysis

We performed three-dimensional structural modeling to assess the binding position of candidate inhibitors to EXO1 using the crystal structure of human wild-type (WT) EXO1 in complex with 5′ recessed-end DNA and Mn^2+^ (PDB: 3QEB). Molecular docking of each compound was accomplished by using the Schrodinger Maestro software. Briefly, the preferred binding site and individual EXO1 residue interactions for the most potent representative compound from each of the seven inhibitor scaffolds were evaluated by performing rigid (Glide) and induced-fit docking for of each compound at the DSP-derived putative inhibitor binding sites, site 1 and site 2. In addition to comparing docking scores to determine their preferred binding site, the hydrogen bonds, salt bridges, and pi stacking interactions were catalogued for each compound to determine which residues may be most critical for inhibitory activity. The residues with the highest number of direct interactions with the inhibitors and/or frequently located within 5 Å of the inhibitors at site 1 and site 2 were selected as candidates for mutagenesis studies.

We further evaluated the docking scores of each of the seven scaffold representatives against X-ray crystallography-resolved structures of the EXO1 protein at nine distinct stages of its exonucleolytic reaction cycle^49^ to determine if our EXO1 inhibitor candidates preferentially bind to a specific conformation of EXO1 (data not shown). For this analysis, we used the following PDB structures: 5UZV, 5V04, 5V05, 5V06, 5V07, 5V08, 5V09, 5V0A, and 5V0B. Docking scores for each compound at site 1 and site 2 were compared, and the preferential binding conformations were defined as those with the most negative docking scores.

### Protein Expression and Purification

The N-terminal domain of human EXO1 (hEXO1) (residues 1–345) was cloned into the pET28b vector. The following mutations were introduced via site-directed mutagenesis based on the findings of molecular docking analysis: K85A, E89A, R92A, I125A, D225A, K292A, and K294A (oligonucleotide primers listed in Table S1) following the established protocol.^50^

Wild-type and mutant hEXO1 N-terminal domains were expressed in E. coli BL21­(DE3) and Rosetta 2­(DE3). The transformed bacteria were then transferred to LB broth (BD Difco) containing kanamycin (100 μg/mL) and shaken continuously (220 rpm) at 37 °C overnight. For large-scale expression, the overnight bacterial growth was adjusted to 0.1 optical density at the 600 nm wavelength (OD600) using kanamycin-containing LB broth and incubated, with continuous shaking, at 37 °C until the culture reached an OD between 0.6 and 0.8. EXO1 expression was then induced by adding 0.4 mM IPTG and placing the mixture in a shaking incubator for 16 h at 16 °C. EXO1-expressing bacteria were then harvested via centrifugation (8000*g*, 4 °C, 15 min) and resuspended in 10 mL of lysis buffer containing 20 mM HEPES-NaOH, pH 7.5; 100 mM NaCl; 20 mM imidazole, pH 8; 5 mM 2-mercaptoethanol (BME); 10% glycerol; 1 mM PMSF; and 1x protease cocktail. The bacterial cells were subsequently lysed by sonication for 10 s (×30 cycles at 50% amplification), the resulting lysate was centrifuged (15,000*g*) at 4 °C for 15 min, and the clear supernatant was isolated for purification. The recombinant EXO1 protein was purified using an KTA Pure chromatography system (Cytiva). The protein sample was loaded into a 1 mL HisTrap Ni-Sepharose column (Cytiva) and equilibrated with a binding buffer containing 20 mM HEPES-NaOH, pH 7.5; 100 mM NaCl; 20 mM imidazole, pH 8; 5 mM BME; and 10% glycerol. The protein-bound column was then washed with 5 column volumes of the binding buffer. A linear gradient of an elution buffer consisting of 20 mM HEPES-NaOH, pH 7.5; 100 mM NaCl; 500 mM imidazole, pH 8; 5 mM BME; and 10% glycerol was used to strip the bound recombinant protein from the column. The eluted fractions containing hEXO1 were identified by using SDS-PAGE and pooled. To ensure that the purity of the protein was suitable for downstream assays, the pooled EXO1-containing sample was diluted with a heparin loading buffer (20 mM HEPES-NaOH, pH 7.5; 100 mM NaCl; 1 mM EDTA; 5 mM BME; and 5% glycerol) and loaded onto a 1 mL heparin column (HiTrap Heparin HP, GE Healthcare Life Sciences), and a linear gradient of heparin elution buffer (20 mM HEPES-NaOH, pH 7.5; 1 M NaCl; 1 mM EDTA; 5 mM BME; and 5% glycerol) was used to strip the bound recombinant protein from the column. The fractions containing pure hEXO1 were identified using SDS-PAGE, and those with ≥90% purity were pooled. The pooled sample was concentrated in a 10,000 MWC Centricon concentrator (Millipore) to a final volume of 0.5 mL. The resulting protein concentrate was aliquoted, flash frozen in dry ice, and immediately stored at – 80 °C for future use. The wild-type human FEN1 enzyme was purified as previously described.^51,52^

### High-Throughput Drug Screening, QC Evaluation, and Data analysis

For high-throughput screening via the FRET-based exonuclease activity assay, 40 nL of compounds from the ChemDiv collection, dissolved in sterile DMSO, was added into empty 384-well black-wall clear-bottom plates using an acoustic dispenser Echo (Beckman Coulter) followed by addition of purified EXO1 protein at 2.2 ng in 30 μL volume per well. After incubation at RT for 20 min, 10 μL of the FAM (6-carboxyfluorescein) fluorophore-labeled DNA substrate was added to the reaction into each well, resulting in the final concentration of substrate at 125 nM and the final compound concentration at 10 μM. Plates were incubated at 37 °C for 30 min, and the fluorescent signal was measured at 485/528 nm using a multimode microplate reader (PerkinElmer Ensight). A final concentration of 10 μM DMSO (0.1% v/v) was included in each screening plate as a negative control. To analyze the high-throughput screening data, the raw fluorescence readouts, along with plate ID, drug concentration, and plate format, were uploaded to CBIS (Cheminnovation.com) for analysis. Quality control (QC) assessment was conducted, which involved calculating the coefficient of variation (CV) of DMSO controls, signal-to-background (S/B) ratio, and assay *Z*’ factor. CV of 3.81% for DMSO, S/B of 8.8, and *Z*’ factor of 0.84 were achieved, which are all well within the industrial standard of <10% of CV, S/B > 4, and *Z*’ factor > 0.5. The percentage of activity for each compound was determined by comparing it to the mean readout of DMSO controls of each plate. Hit selection thresholds were set at >50% inhibition compared to DMSO control, and 84 preliminary hits were selected.

### Hit Confirmation, Validation, and Counter Screening

For subsequent hit confirmation, 84 compounds from the primary screen were picked from our internal stock and subjected to a dose-dependent response assay starting at 100 μM with 3-fold serial dilution. To analyze the data from the dose-dependent response assay, IC_50_ values for each hit were calculated through nonlinear regression analysis using GraphPad Prism 10 (GraphPad Software, Inc.). Of the initial 84 compounds, 20 compounds demonstrated IC_50_ values less than 20 μM and were isolated for further validation. Subsequently, a counter screen was performed to eliminate potential false-positive hits. To accomplish this, we established an analogous FRET assay using the purified FEN1 enzyme in place of EXO1. To be considered a hit compound, IC_50_ values in the EXO1 assay were required to be less than 20 μM, and the IC_50_ ratio between FEN1 and EXO1 must be more than 5-fold.

### Immunofluorescence Staining

Cells were seeded at a density of 3× 10^5^ cells per well in 12-well plates with preplaced coverslips and treated with or without various inhibitor treatments overnight. Cells were then incubated with EdU for 30 min before collection. For PARylation analysis, cells were permeabilized with 0.1% Triton X100, fixed with 4% paraformaldehyde, blocked with the Image-iT FX signal enhancer (Invitrogen), and incubated (1.5 h, RT) with the indicated primary antibodies (PAR/pADPr Antibody; Biotechne). The cells were then washed with PBS buffer and incubated (1 h, RT) with the corresponding secondary antibodies (1:200, Invitrogen). Subsequently, the fixed cells were incubated with the Click-iT reaction mix (100 mM Tris-HCl pH 8.5, 10 mM azide-Alexa 488, 1 mM CuSO4, and 100 mM ascorbic acid (Sigma-Aldrich)) for 1 h at RT. The slides were then washed with the PBS buffer, counterstained with DAPI, and analyzed with a fluorescence microscope (Zeiss Observer Z1). For γ-H2AX staining, cells were fixed with 4% paraformaldehyde, blocked with the Image-iT FX signal enhancer (Invitrogen), and incubated (1.5 h, RT) with the indicated primary antibodies (γ-H2AX). The cells were then washed with the PBS buffer and incubated (1 h, RT) with the corresponding secondary antibodies (1:200, Invitrogen). The slides were then washed with the PBS buffer, incubated with the Click-iT reaction mix for EdU as aforementioned, counterstained with DAPI, and analyzed with a fluorescence microscope (Zeiss Observer Z1).

### Gel-Based Phospho-RPA End Resection Assay

To examine the impact of EXO1 inhibition on DSB end resection, MDA-MB-231 cells were treated with 0, 20, or 40 μM compound C200 or F684 overnight and subsequently exposed to no camptothecin (CPT) or 2 μM CPT for 2 h to cause replication fork stalling and induce DNA single-strand breaks (SSBs) that progress to DSBs in the S-phase. The treated cells were then lysed in the SDS-PAGE sample buffer, and the lysate was separated by SDS-PAGE prior to immobilization on a nitrocellulose membrane and Western blotting. Antibodies against unphosphorylated RPA, S33-P-RPA2, and tubulin were subsequently assayed according to the manufacturer’s recommendations. The ratio of P-RPA/RPA levels were quantified via ImageJ.

### Homologous Recombination Assay

To evaluate the effects of C200 and F684 on HR, U2OS cells stably expressing an HR-GFP reporter were utilized as previously described.^53,54^ Cells were infected with an adenovirus expressing the I-SceI restriction enzyme for 16 h to induce DNA DSBs. Following viral infection, the culture medium was replaced with a fresh medium containing small molecule inhibitors. Cells were treated with 0, 10, 50, or 100 μM C200 or F684, either alone or in combination with the DNA2 inhibitor C5 (10 μM), and incubated for an additional 48 h. Subsequently, cells were harvested, and GFP-positive frequencies indicative of HR repair events were quantified using a CyAn ADP Analyzer (Beckman Coulter, Inc.). Experiments were performed in triplicate, and data are presented as mean ± standard deviation (S.D.).

### National Cancer Institute Human Tumor Cell Line Drug Profiling

We tested the cytotoxicity of representative compounds from the seven EXO1i scaffolds against a subset of the NCI-60 cell line panel along with three additional pancreatic cell lines (FG, AsPC1, and CaPan2). In general, cells were seeded in 384-well tissue culture-treated plates overnight and treated with various concentrations of each hit compound (a serial 3-fold dilution from 50 μM for all tested hits). Each concentration was performed in duplicate. Cell viability was then measured by CellTiter Glo (Promega) after 72 h of treatment. The IC_50_, defined as the drug concentration that decreased viability by 50%, was calculated using nonlinear regression analysis via Prism 10 (GraphPad Software, Inc.).

### Annexin V Apoptosis Assay

MDA-MB-436 parental cells (BRCA1−) and MDA-MB-436 expressing WT BRCA1 (BRCA1+) were seeded into 384-well clear-bottom black plates (Cat. #781090, Greiner Bio-One, USA) at a density of 1500 cells per well in 30 μL of the RPMI-1640 medium followed by incubating at 37 °C and 5% CO_2_. After 24 h of incubation, testing compounds were added to cells at final concentrations of 50, 25, 12, and 5 μM using the Echo Liquid Handler (Beckman). Subsequently, 10 μL of the NIR-labeled Annexin V apoptosis reagent (Cat. #4768, Sartorius, Germany) was added to each well at a 1:200 final dilution. The plates were then transferred to the Incucyte Live-Cell Analysis System for real-time apoptosis monitoring over a 5 day period. Imaging was conducted using a 10× objective with phase-contrast and near-infrared (NIR) fluorescent channels, capturing scans every 2 h. Image analysis was performed with the Incucyte software, and the total NIR fluorescence intensity was quantified to evaluate the apoptosis.

### *In* *Vitro* Cell Survival Assay

The survival rates of MDA-MB-436 parental cells (BRCA1−) and WT BRCA1 expressing MDA-MB-436 (BRCA1+) cells were assessed by cell counting. For this assay, 10,000 cells were seeded per well in a 24-well plate and incubated in a complete culture medium containing DMSO or the EXO1i of interest at 37 °C with 5% CO_2_. The medium was refreshed every 3 to 4 days by replacing it with a fresh medium containing DMSO or EXO1i. After 5 days, the cells were trypsinized, and the survival curve was generated by manual cell counting.

### *In* *Vivo* Tumor Growth Assay

The tumor growth rate of human breast cancer cells with or without treatment with EXO1i was evaluated by using a xenograft breast cancer mouse model. Six to eight week old male or female NSG (NOD SCID gamma) mice were grafted with 4,000,000 MDA-MB-436 (BRCA1−) or MDA-MB-436 (BRCA1+) cells per mouse. EXO1 inhibitor F684 was started once the tumor volume reached 100 mm^3^. The vehicle control or F684 (8.5 mg/kg body weight) was administered daily via intraperitoneal injection. The tumor volume in each mouse was measured twice a week and calculated using the formula (length × width × width)/2 (mm^3^). When the tumors reached a volume of 1000 mm^3^, the mice were euthanized. Animal studies were conducted in accordance with an approved protocol adhering to the IACUC policies and procedures of the City of Hope.

### Statistical Analysis

All experimental values are presented as the average ± standard deviation of at least three technical replicates for three independent experiments, unless indicated otherwise. In cell-based assays, statistical significance for each experimental condition was assessed by comparison to DMSO-treated (vehicle control) samples via Student’s *t* test. Sample groups with *p* < 0.05 were deemed significantly different from DMSO controls.

## Supplementary Material



## Notes

Additional experimental details and materials, including assay validations, counter screening data against FEN1, and oligonucleotide primers and molecular docking models used in mutagenesis studies (PDF)

## +

Department of Gastroenterology, Tongren Hospital, Shanghai Jiao Tong University School of Medicine, Shanghai 200231, China

## >

Department of Neurosurgery, Stanford University, Stanford, California 94305, USA

## <

Department of Biological Chemistry, University of California Los Angeles, Los Angeles, California 90095, USA

## References

[1] Bartkova J., Rezaei N., Liontos M. (2006). Oncogene-induced senescence
is part of the tumorigenesis barrier imposed by DNA damage checkpoints. Nature.

[2] Negrini S., Gorgoulis V. G., Halazonetis T. D. (2010). Genomic instability--an evolving
hallmark of cancer. Nat. Rev. Mol. Cell Biol..

[3] Zheng L., Dai H., Zhou M. (2012). Polyploid cells rewire DNA damage response
networks to overcome replication stress-induced barriers for tumour
progression. Nat. Commun..

[4] Gaillard H., Garcia-Muse T., Aguilera A. (2015). Replication stress and cancer. Nature reviews Cancer.

[5] Macheret M., Halazonetis T. D. (2015). DNA replication
stress as a hallmark of cancer. Annual review
of pathology.

[6] Bennett C. B., Westmoreland T. J., Snipe J. R., Resnick M. A. (1996). A double-strand
break within a yeast artificial chromosome (YAC) containing human
DNA can result in YAC loss, deletion or cell lethality. Mol. Cell. Biol..

[7] Bennett C. B., Lewis A. L., Baldwin K. K., Resnick M. A. (1993). Lethality induced
by a single site-specific double-strand break in a dispensable yeast
plasmid. Proc. Natl. Acad. Sci. U. S. A..

[8] Cannan W. J., Pederson D. S. (2016). Mechanisms and Consequences of Double-Strand DNA Break
Formation in Chromatin. J. Cell Physiol.

[9] Helleday T., Petermann E., Lundin C., Hodgson B., Sharma R. A. (2008). DNA repair
pathways as targets for cancer therapy. Nat.
Rev. Cancer.

[10] Somaiah N., Yarnold J., Daley F. (2012). The relationship between
homologous recombination repair and the sensitivity of human epidermis
to the size of daily doses over a 5-week course of breast radiotherapy. Clin. Cancer Res..

[11] Rajamanickam S., Park J. H., Subbarayalu P. (2022). Targeting aberrant replication
and DNA repair events for treating breast cancers. Commun. Biol..

[12] Genschel J., Modrich P. (2003). Mechanism of 5′-Directed
Excision in Human Mismatch
Repair. Mol. Cell.

[13] Fiorentini P., Huang K. N., Tishkoff D. X., Kolodner R. D., Symington L. S. (1997). Exonuclease
I of Saccharomyces cerevisiae functions in mitotic recombination in
vivo and in vitro. Mol. Cell. Biol..

[14] Maringele L., Lydall D. (2002). EXO1-dependent single-stranded DNA at telomeres activates
subsets of DNA damage and spindle checkpoint pathways in budding yeast
yku70Delta mutants. Genes Dev..

[15] Cotta-Ramusino C., Fachinetti D., Lucca C. (2005). Exo1 processes stalled
replication forks and counteracts fork reversal in checkpoint-defective
cells. Mol. Cell.

[16] Zheng L., Jia J., Finger L. D., Guo Z., Zer C., Shen B. (2011). Functional
regulation of FEN1 nuclease and its link to cancer. Nucleic Acids Res..

[17] Zheng L., Meng Y., Campbell J. L., Shen B. (2020). Multiple roles of DNA2
nuclease/helicase in DNA metabolism, genome stability and human diseases. Nucleic Acids Res..

[18] Zheng L., Shen B. (2011). Okazaki fragment maturation:
nucleases take centre stage. J. Mol. Cell Biol..

[19] Zimmermann M., Murina O., Reijns M. A. M. (2018). CRISPR screens identify
genomic ribonucleotides as a source of PARP-trapping lesions. Nature.

[20] Nimonkar A. V., Genschel J., Kinoshita E. (2011). BLM-DNA2-RPA-MRN and
EXO1-BLM-RPA-MRN constitute two DNA end resection machineries for
human DNA break repair. Genes Dev..

[21] Bao S., Tibbetts R. S., Brumbaugh K. M. (2001). ATR/ATM-mediated phosphorylation
of human Rad17 is required for genotoxic stress responses. Nature.

[22] Cortez D., Guntuku S., Qin J., Elledge S. J. (2001). ATR and ATRIP: partners
in checkpoint signaling. Science.

[23] Zou L., Elledge S. J. (2003). Sensing
DNA damage through ATRIP recognition of RPA-ssDNA
complexes. Science.

[24] Ira G., Pellicioli A., Balijja A. (2004). DNA end resection, homologous
recombination and DNA damage checkpoint activation require CDK1. Nature.

[25] Kakarougkas A., Jeggo P. A. (2014). DNA DSB repair pathway
choice: an orchestrated handover
mechanism. British journal of radiology.

[26] Hale A., Dhoonmoon A., Straka J., Nicolae C. M., Moldovan G. L. (2023). Multi-step
processing of replication stress-derived nascent strand DNA gaps by
MRE11 and EXO1 nucleases. Nat. Commun..

[27] Somaiah N., Yarnold J., Daley F. (2012). The relationship between
homologous recombination repair and the sensitivity of human epidermis
to the size of daily doses over a 5-week course of breast radiotherapy. Clinical Cancer Research: an Official Journal of the American
Association For Cancer Research.

[28] Caldon C. E. (2014). Estrogen
signaling and the DNA damage response in hormone dependent breast
cancers. Front. Oncol..

[29] Lord C. J., Ashworth A. (2017). PARP inhibitors: Synthetic
lethality in the clinic. Science (New York,
NY).

[30] Cong K., Peng M., Kousholt A. N. (2021). Replication gaps are
a key determinant of PARP inhibitor synthetic lethality with BRCA
deficiency. Mol. Cell.

[31] Farmer H., McCabe N., Lord C. J. (2005). Targeting the DNA repair
defect in BRCA mutant cells as a therapeutic strategy. Nature.

[32] Bolderson E., Tomimatsu N., Richard D. J. (2010). Phosphorylation of Exo1
modulates homologous recombination repair of DNA double-strand breaks. Nucleic Acids Res..

[33] Paiano J., Zolnerowich N., Wu W. (2021). Role of 53BP1 in end
protection and DNA synthesis at DNA breaks. Genes Dev..

[34] Michl J., Zimmer J., Buffa F. M., McDermott U., Tarsounas M. (2016). FANCD2 limits replication stress and genome instability
in cells lacking BRCA2. Nat. Struct Mol. Biol..

[35] Liu W., Zhou M., Li Z. (2016). A Selective Small Molecule
DNA2 Inhibitor for Sensitization of Human Cancer Cells to Chemotherapy. EBioMedicine.

[36] Qiu J., Qian Y., Chen V., Guan M. X., Shen B. (1999). Human exonuclease
1 functionally complements its yeast homologues in DNA recombination,
RNA primer removal, and mutation avoidance. J. Biol. Chem..

[37] Grasby J. A., Finger L. D., Tsutakawa S. E., Atack J. M., Tainer J. A. (2012). Unpairing
and gating: sequence-independent substrate recognition by FEN superfamily
nucleases. Trends Biochem. Sci..

[38] Casu B., Arya T., Bessette B., Baron C. (2017). Fragment-based screening
identifies novel targets for inhibitors of conjugative transfer of
antimicrobial resistance by plasmid pKM101. Sci. Rep..

[39] Kuo L. J., Yang L. X. (2008). Gamma-H2AX - a novel
biomarker for DNA double-strand
breaks. In Vivo.

[40] Orans J., McSweeney E. A., Iyer R. R. (2011). Structures of human
exonuclease 1 DNA complexes suggest a unified mechanism for nuclease
family. Cell.

[41] van
de Kooij B., Schreuder A., Pavani R. (2024). EXO1 protects
BRCA1-deficient cells against toxic DNA lesions. Mol. Cell.

[42] García-Rodríguez N., Domínguez-García I., Domínguez-Pérez M. D. C., Huertas P. (2024). EXO1 and DNA2-mediated ssDNA gap expansion is essential
for ATR activation and to maintain viability in BRCA1-deficient cells. Nucleic Acids Res..

[43] Bryant H. E., Schultz N., Thomas H. D. (2005). Specific killing of
BRCA2-deficient tumours with inhibitors of poly­(ADP-ribose) polymerase. Nature.

[44] Chappidi N., De Gregorio G., Ferrari S. (2019). Replication stress-induced Exo1 phosphorylation
is mediated by Rad53/Pph3 and Exo1 nuclear localization is controlled
by 14–3-3 proteins. Cell Div..

[45] Daina A., Michielin O., Zoete V. (2017). SwissADME: a free web
tool to evaluate
pharmacokinetics, drug-likeness and medicinal chemistry friendliness
of small molecules. Sci. Rep..

[46] Ritchie T. J., Ertl P., Lewis R. (2011). The graphical representation of ADME-related
molecule properties for medicinal chemists. Drug Discov Today.

[47] Lovering F., Bikker J., Humblet C. (2009). Escape from
flatland: increasing
saturation as an approach to improving clinical success. J. Med. Chem..

[48] Lipinski C. A., Lombardo F., Dominy B. W., Feeney P. J. (2001). Experimental and
computational approaches to estimate solubility and permeability in
drug discovery and development settings. Adv.
Drug Deliv Rev..

[49] Shi Y., Hellinga H. W., Beese L. S. (2017). Interplay
of catalysis, fidelity,
threading, and processivity in the exo- and endonucleolytic reactions
of human exonuclease I. Proc. Natl. Acad. Sci.
U. S. A..

[50] Sun X., Zheng L., Shen B. (2002). Functional alterations of human exonuclease
1 mutants identified in atypical hereditary nonpolyposis colorectal
cancer syndrome. Cancer Res..

[51] Sridharan M., Battapadi T., Balakrishnan L. (2024). Affinity Purification of a 6X-His-Tagged
Protein using a Fast Protein Liquid Chromatography System. J. Visualized Exp..

[52] Shen B., Nolan J. P., Sklar L. A., Park M. S. (1996). Essential amino
acids for substrate binding and catalysis of human flap endonuclease
1. J. Biol. Chem..

[53] Gunn A., Stark J. M. (2012). I-SceI-based assays
to examine distinct repair outcomes
of mammalian chromosomal double strand breaks. Methods Mol. Biol..

[54] Yang L., Shen C., Estrada-Bernal A. (2021). Oncogenic KRAS drives
radioresistance through upregulation of NRF2–53BP1-mediated
non-homologous end-joining repair. Nucleic Acids
Res..

