# Supplementary material for: Discovery and Characterization of Small Molecule Inhibitors Targeting Exonuclease 1 for Homologous Recombination-Deficient Cancer Therapy
Source: ACS Chem Biol. 2025 May 16;20(6):1258–72. doi: 10.1021/acschembio.5c00117 (PMC12186255; doi:10.1021/acschembio.5c00117)
Supplement: Supplementary file 1 [file cb5c00117_si_001.pdf]

Supporting Information

Discovery and characterization of small molecule inhibitors targeting exonuclease 1 for homologous recombination-deficient cancer therapy

Yixing Wang, Jessica D. Hess, Chen Wang, Lingzi Ma, Megan Luo, Jennifer Jossart, John J. Perry, David Kwon, Zhe Wang, Xinyu Pei, Changxian Shen, Yingying Wang, Mian Zhou, Holly Yin, David Horne, André Nussenzweig, Li Zheng, and Binghui Shen

Supplementary Tables

Table S1. Oligonucleotide primers used in mutagenesis studies

Table S2. Docking scores (kcal/mol) of C200 against EXO1 mutants

Table S3. NCI-60 cell line panel IC_{50}s

Supplementary Figures

Figure S1. High-throughput screening assay validation.

Figure S2. Three-dimensional structure of EXO1 vs FEN1 N-terminal domains.

Figure S3. IC_{50} of inhibitor scaffolds against FEN1 protein via FRET-based HTS assay.

Figure S4. Binding of EXO1 inhibitor scaffolds via thermal shift assay.

Figure S5. Inhibitor kinetics for compound F684.

Figure S6. Inhibitor binding pockets of EXO1.

Figure S7. Purified EXO1 mutants.

Figure S8. Binding of inhibitors within the Site 1 and Site 2 binding pockets.

Figure S9. Effect of Site 1 point mutations on C200 binding position.

Figure S10. Positional comparison of C200 binding before and after Site 1 point mutations.

Figure S11. Effect of Site 2 point mutations on C200 binding position.

Figure S12. Positional comparison of C200 binding after Site 2 point mutations.

Figure S13. Effect of EXO1 point mutations on enzyme activity.

Table S1. Oligonucleotide primers used in mutagenesis studies

Mutation	Oligonucleotides
Lys85Ala F	5’-TCTTCTAGATCTCTCTACTTCCTTTGCAGAAGGTAAAGTACATCCATCAAAT-3’
Lys85Ala R	5’-ATTTGATGGATGTACTTTACCTTCTGCAAAGGAAGTAGAGAGATCTAGAAGA-3’
Glu89Ala F	5’-CGTCTTTCTCTTCTAGATCTCGCTACTTCCTTTTTAGAAGGTA-3’
Glu89Ala R	5’-TACCTTCTAAAAAGGAAGTAGCGAGATCTAGAAGAGAAAGACG-3’
Arg92Ala F	5’-GATTGGCTTGTCGTCTTTCTCTTGCAGATCTCTCTACTTCCTTTTTAG-3’
Arg92Ala R	5’-CTAAAAAGGAAGTAGAGAGATCTGCAAGAGAAAGACGACAAGCCAATC-3’
Ile125Ala F	5’-GGGCCATGGCATGTGTGGCATTGATAGACCGGGTGAA-3’
Ile125Ala R	5’-TTCACCCGGTCTATCAATGCCACACATGCCATGGCCC-3’
Asp225Ala F	5’-CCCGGTCTATCAATGCCACACATGCCATGG-3’
Asp225Ala R	5’-CCATGGCATGTGTGGCATTGATAGACCGGG-3’
Lys292Ala/ Lys294Ala F	5’-CTTCATAGGCGTTCAGAGGAATAAGTGCCCTTGCGATGGGATCAAAAACTAGCTGATAGA-3’
Lys292Ala/ Lys294Ala R	5’-TCTATCAGCTAGTTTTTGATCCCATCGCAAGGGCACTTATTCCTCTGAACGCCTATGAAG-3’

Table S2. Docking scores (kcal/mol) of C200 against EXO1 mutants

C200	Rigid Score	Induced-Fit Score
Site 1		
WT	-6.620	-781.70
K85A	-7.489	-15691.59
E89A	-6.777	-761.33
R92A*	-5.531	-785.21
I125A	-6.544	-15615.61
D225A*	-5.491	-15754.60
Site 2		
WT	-2.334	-770.85
K292A/K294A*	-2.364	-770.55
**F684**	**Rigid Score**	**Induced-Fit Score**
Site 1		
WT	-11.448	-15218.55
K85A*	-8.900	-15091.50
E89A	-9.775	-15080.40
R92A*	-10.375	-15091.14
I125A	-11.110	-15155.45
D225A	-11.145	-15100.80
Site 2		
WT	-5.209	-747.77
K292A/K294A	-4.066	-741.35

Table S3. NCI-60 cell line panel IC_{50}s

	F684-0064	C200	D351-0317	D351-0328	D413-0291	D413-0213	F685-0020	G889-0100	G889-0101	G889-0196	C73
Breast											
BT549	11.95	>50.00	8.64	9.76	23.37	23.82	17.88	31.48	37.81	>50.00	1.73
T-47D	17.97	>50.00	13.91	13.72	13.70	16.42	16.86	36.25	25.39	30.78	13.76
HS 578T	18.19	>50.00	17.57	15.33	15.45	20.11	22.93	>50.00	19.92	>50.00	5.29
MCF7	20.08	>50.00	43.29	27.24	18.02	37.79	43.52	>50.00	43.56	>50.00	4.50
MCF7 (B3)	23.59	>50.00	28.25	28.44	21.43	25.17	37.27	>50.00	37.46	>50.00	3.84
MDA-MB-468	23.62	>50.00	40.46	25.86	27.65	20.00	46.35	>50.00	>50.00	>50.00	0.83
MDA-MB-231	28.56	>50.00	19.66	21.50	16.05	21.29	31.59	>50.00	35.19	>50.00	2.27
Ovarian											
OVCAR-3	10.93	>50.00	7.59	7.38	9.76	10.97	5.73	18.69	10.85	31.06	2.18
OVCAR-4	15.38	>50.00	10.29	7.14	15.38	23.36	21.42	31.74	21.54	>50.00	24.97
IGR-OV1	16.24	>50.00	12.99	12.51	9.28	14.95	18.09	34.21	29.49	>50.00	2.13
OVCAR-8	18.12	>50.00	10.80	14.24	8.51	13.63	18.77	>50.00	16.30	>50.00	1.88
SKOV-3	18.46	>50.00	19.28	17.16	20.95	18.86	24.36	>50.00	46.46	>50.00	24.68
NCI/ADR-RES	20.83	>50.00	15.44	17.94	16.86	19.14	17.70	>50.00	17.23	37.73	1.91
OVCAR-5	21.58	>50.00	18.51	17.29	21.60	21.38	19.36	48.59	29.17	47.27	1.75
Prostate											
DU145	16.72	49.37	12.83	11.52	18.03	15.96	12.57	27.99	35.93	>50.00	2.42
DU145 (B3)	15.38	>50.00	27.12	21.32	16.69	16.85	14.09	33.30	24.14	>50.00	1.09
PC3	18.02	>50.00	14.13	14.53	14.28	19.77	19.74	41.72	26.58	>50.00	6.60
Leukemia											
CCRF-CEM	14.64	27.40	10.32	7.02	9.16	9.84	14.82	34.69	8.14	15.32	0.61
HL-60(TB)	20.10	>50.00	12.17	14.03	15.87	18.05	17.47	43.15	28.45	46.78	0.76
Melanoma											
UACC-257	24.96	>50.00	20.48	16.07	19.22	20.77	21.46	50.27	49.56	>50.00	1.47
SK-MEL-28	37.96	>50.00	16.52	15.11	19.05	20.36	47.14	>50.00	>50.00	>50.00	6.10
Pancreatic											
FG	27.54	>50.00	43.09	>50.00	20.75	25.52	>50.00	>50.00	>50.00	>50.00	5.34
CaPan2	48.09	>50.00	30.22	28.15	22.47	23.64	44.69	>50.00	>50.00	>50.00	12.47
AsPC1	>50.00	>50.00	43.44	25.38	22.79	23.80	43.28	>50.00	27.04	>50.00	4.11
Central Nervous System											
U251	16.17	>50.00	9.79	9.64	9.34	13.64	14.22	31.67	30.21	40.68	0.77
SNB-19	19.34	>50.00	20.79	17.97	19.90	19.54	16.64	>50.00	41.54	>50.00	3.77
SF-295	26.98	>50.00	39.60	>50.00	38.13	40.01	28.21	>50.00	>50.00	>50.00	1.70
SF-268	38.62	>50.00	40.98	42.51	38.19	38.29	49.29	>50.00	>50.00	>50.00	2.47
Lung											
H23	23.39	>50.00	43.06	44.56	17.11	19.54	23.48	>50.00	>50.00	>50.00	2.89
Kidney											
UO-31	20.56	>50.00	9.20	9.96	13.93	16.82	8.27	34.57	15.70	>50.00	0.81
ACHN	28.91	>50.00	19.19	19.31	16.24	21.28	19.87	>50.00	42.01	>50.00	1.26

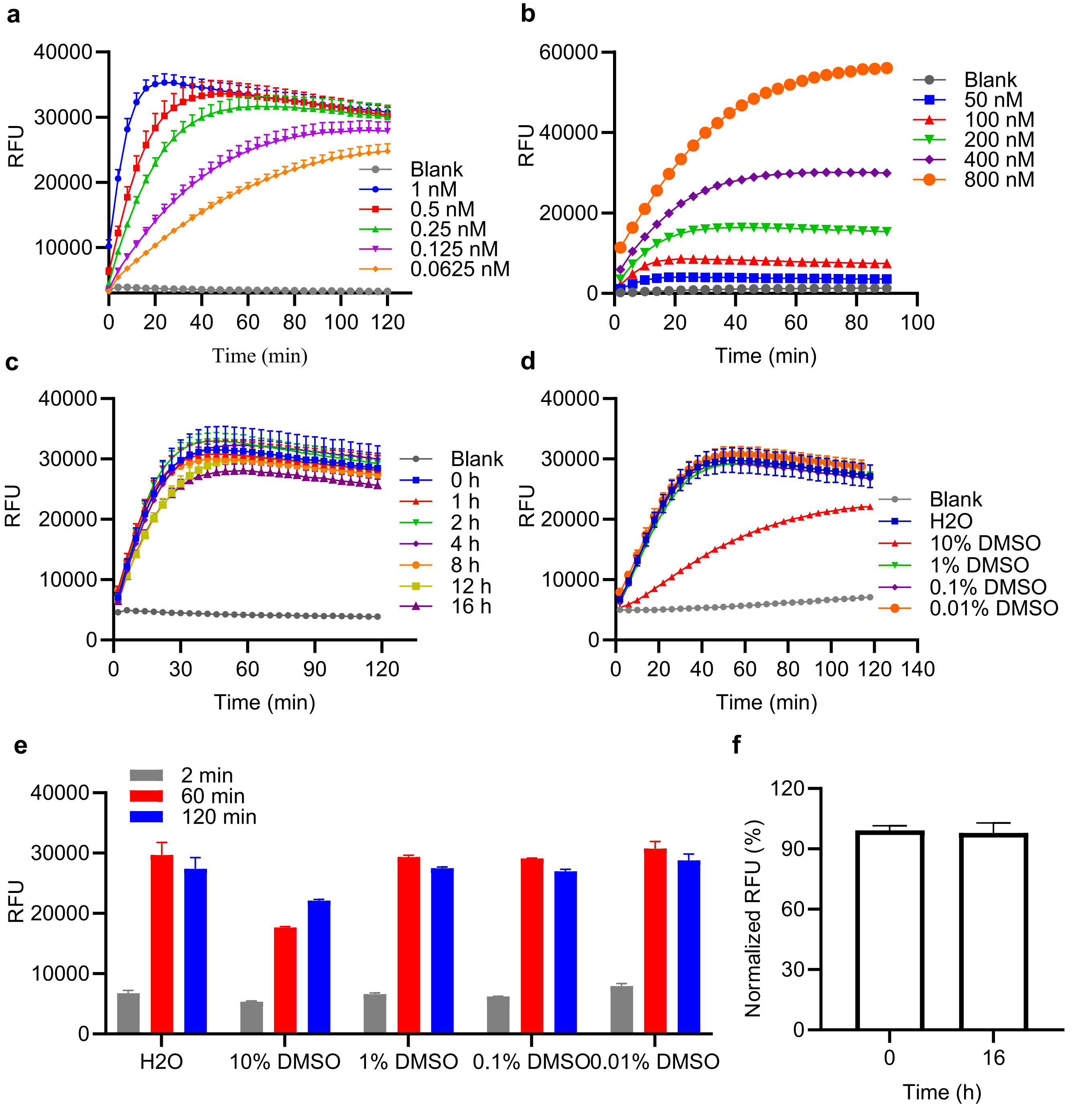

Figure S1. High-throughput screening assay validation.
(a) Time course of RFU in reactions with 100 nM DNA substrates and varying conc. of EXO1. Values are mean ± s.d. of three independent assays. **(b)** EXO1 activity in the presence of 100 pM EXO and varying conc. of DNA substrates. Values are mean ± s.d. of three independent assays. **(c)** Stability of EXO1 activity over time. EXO1 protein in reaction buffer was kept on ice for 0–16 h. Time-dependent EXO1 activities of the variously incubated samples were monitored. Values are mean ± s.d. of three independent assays. **(d)** Time course of RFU in reactions with varying conc. of DMSO. Values are mean ± s.d. of three independent assays. **(e)** Normalized RFU in the reaction with or without the addition of varying concentrations of DMSO. Values are mean ± s.d. of three independent assays. **(f)** Stability of EXO1 protein. EXO1 (concentrated) in stock buffer was maintained at room temperature for 16 h, then EXO1 nuclease activity was assayed using the FRET-based assay, comparing 16 h vs. 0 h.

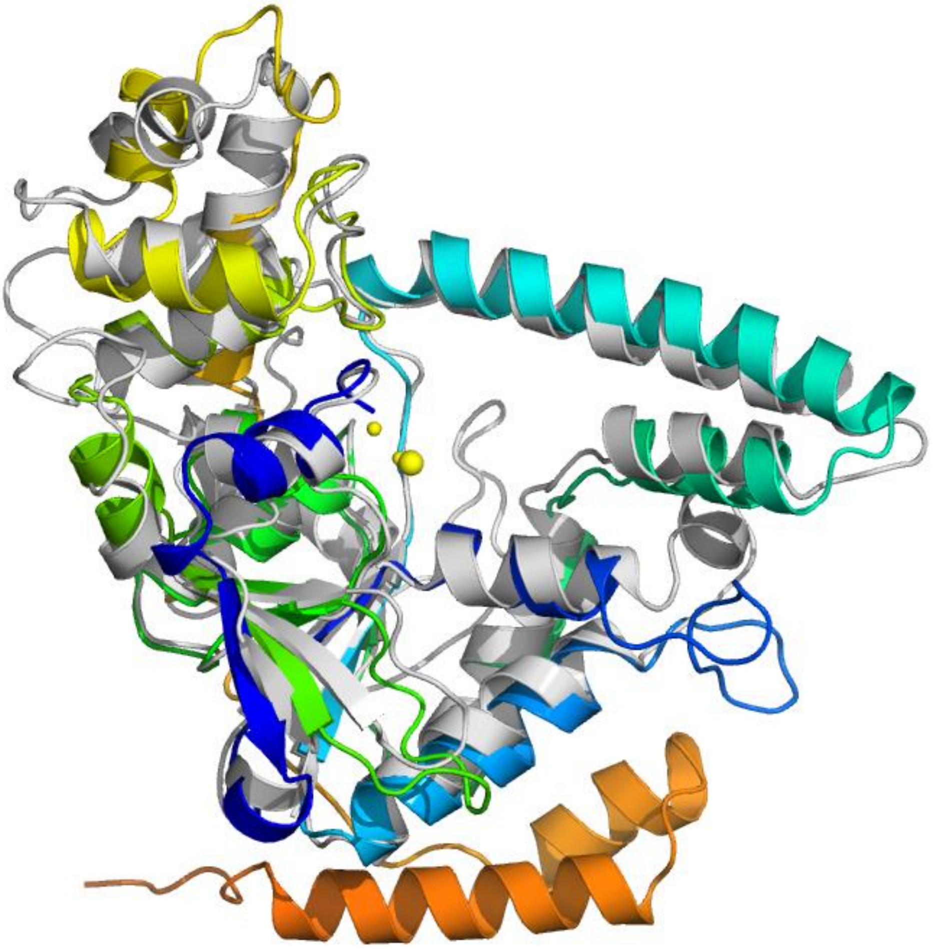

Figure S2. Three-dimensional structure of EXO1 vs FEN1 N-terminal domains.
Comparison of the N-terminal domains of human EXO1 (grey; PDB 5UZV) and FEN1 (multicolor; PDB 3Q8K) proteins, highlighting the significant overlap of their three-dimensional structure (29% sequence identity). Structural alignment and visualization were performed using Maestro (Schrödinger). Yellow circles indicate the location of catalytic metal ions within their respective active sites.

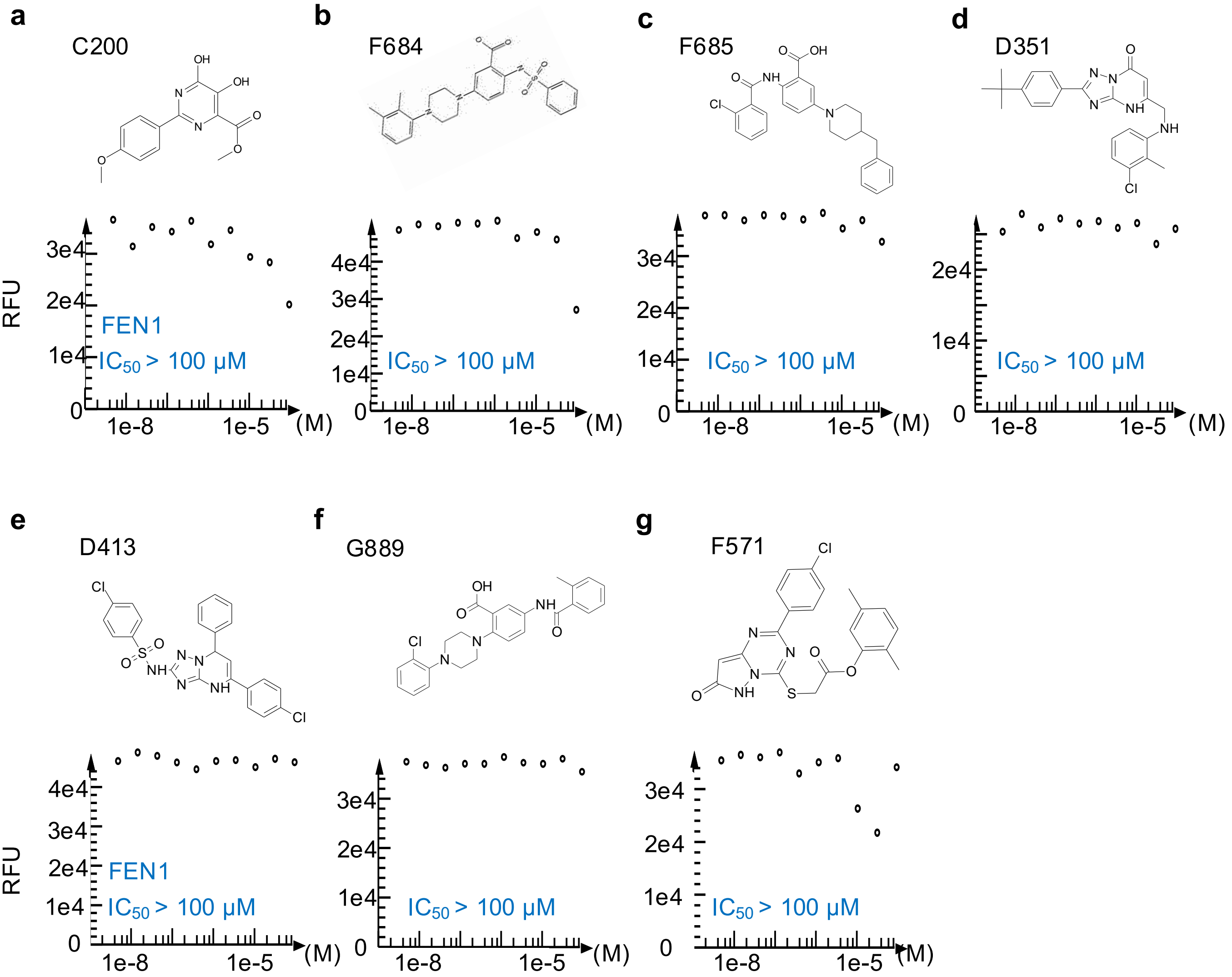

Figure S3. IC_{50} of inhibitor scaffolds against FEN1 protein via FRET-based HTS assay.
(a-g) Dose-response curves and calculated IC_{50}s of the seven EXO1 inhibitor scaffolds against the closely related protein family member FEN1. A maximum inhibitor concentration of 100 μM was tested. IC_{50} values for each hit were calculated through nonlinear regression analysis.

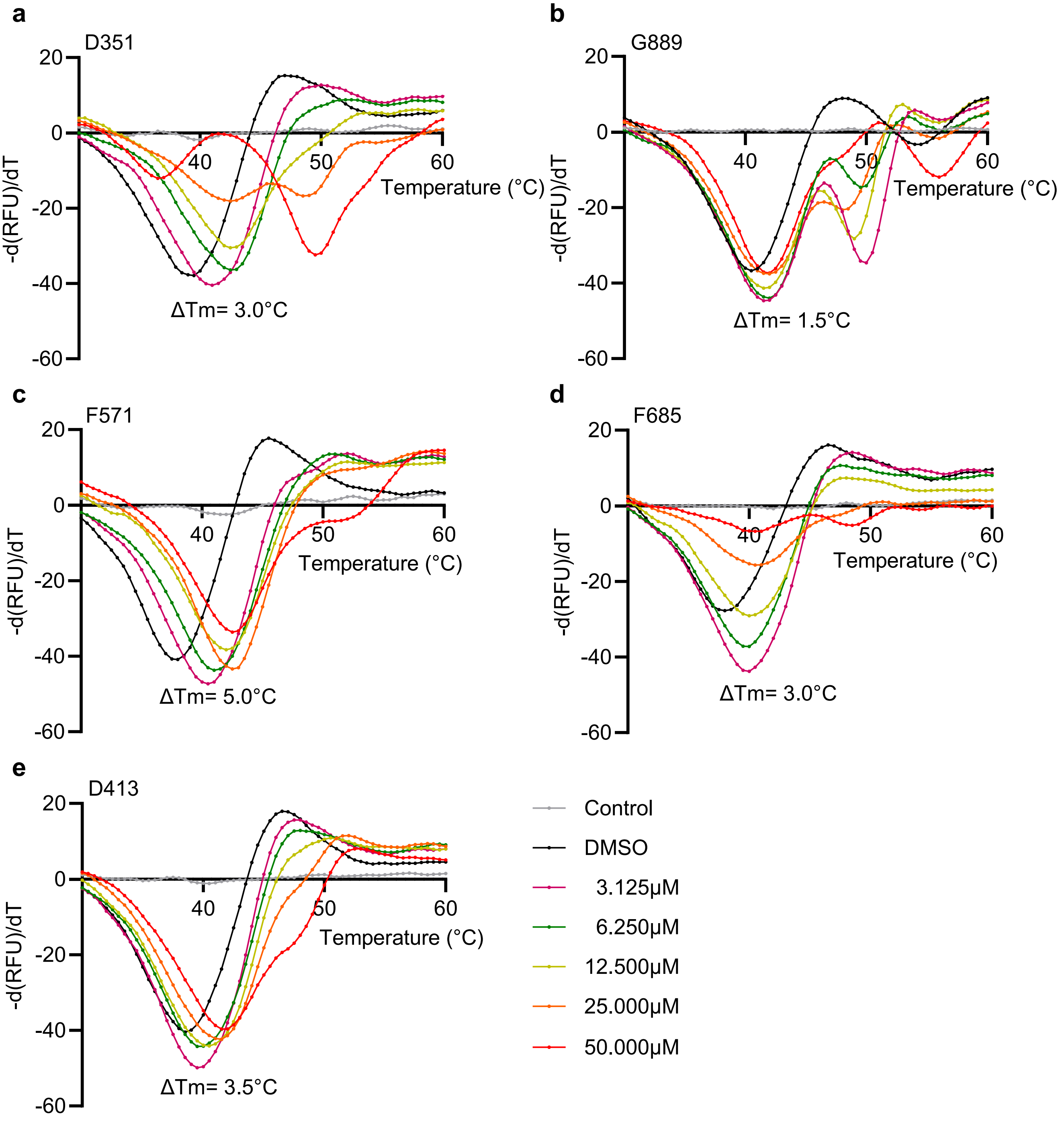

Figure S4. Binding of EXO1 inhibitor scaffolds via thermal shift assay.
Derivative of thermal shift assay melt curves for compound D351 (ΔT_m 3.0 ºC) **(a)**, G889 (ΔT_m 1.5 ºC) **(b)**, F571 (ΔT_m 5.0 ºC) **(c)**, F685 (ΔT_m 3.0 ºC) **(d)**, and D413 (ΔT_m 3.5 ºC) **(e)**. In each experiment, baseline protein melting temperature was evaluated using an untreated control containing only protein and dye, and thermal shifts were measured by comparing the change in T_m between vehicle (DMSO) and experimental compound treatments.

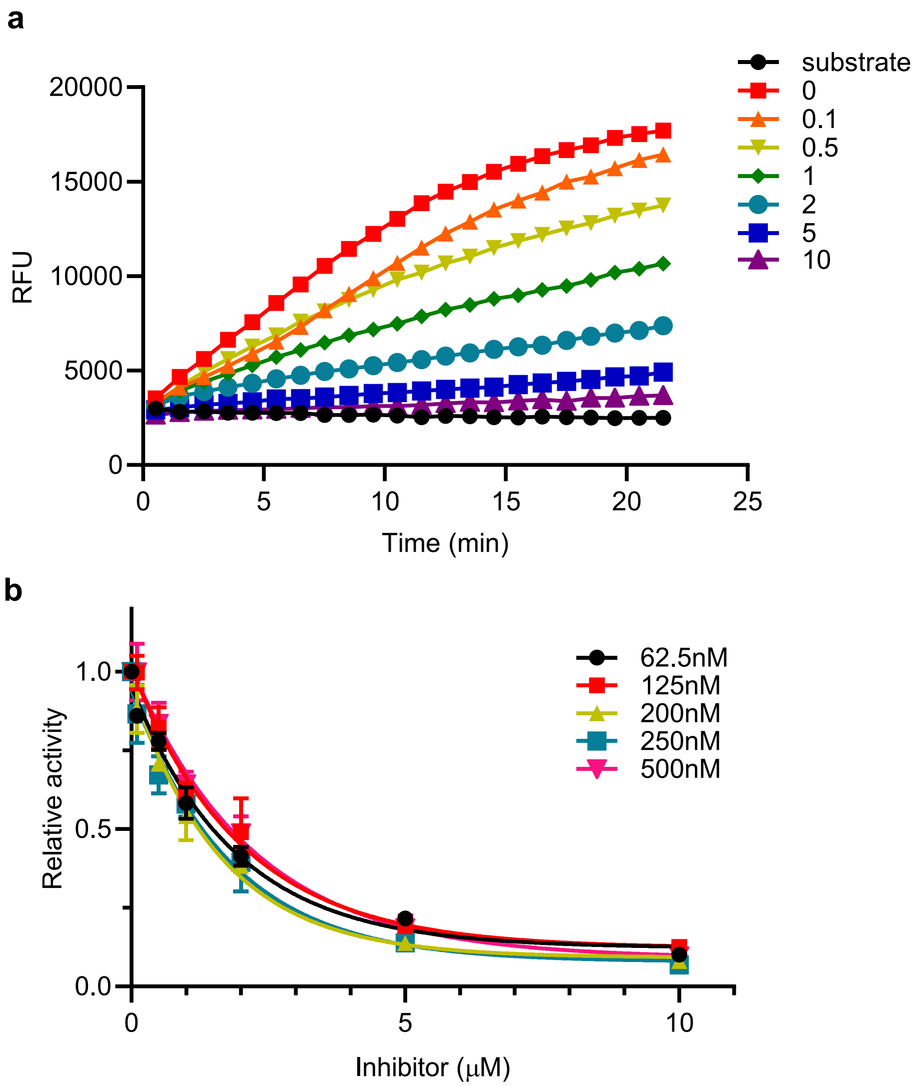

Figure S5. Inhibitor kinetics for compound F684.
(a) Time course of EXO1 exonuclease reaction with varying concentrations of EXO1 inhibitor F684. **(b)** Relative exonuclease activity of EXO1 with varying concentrations of DNA substrate and inhibitor F684.

a

b

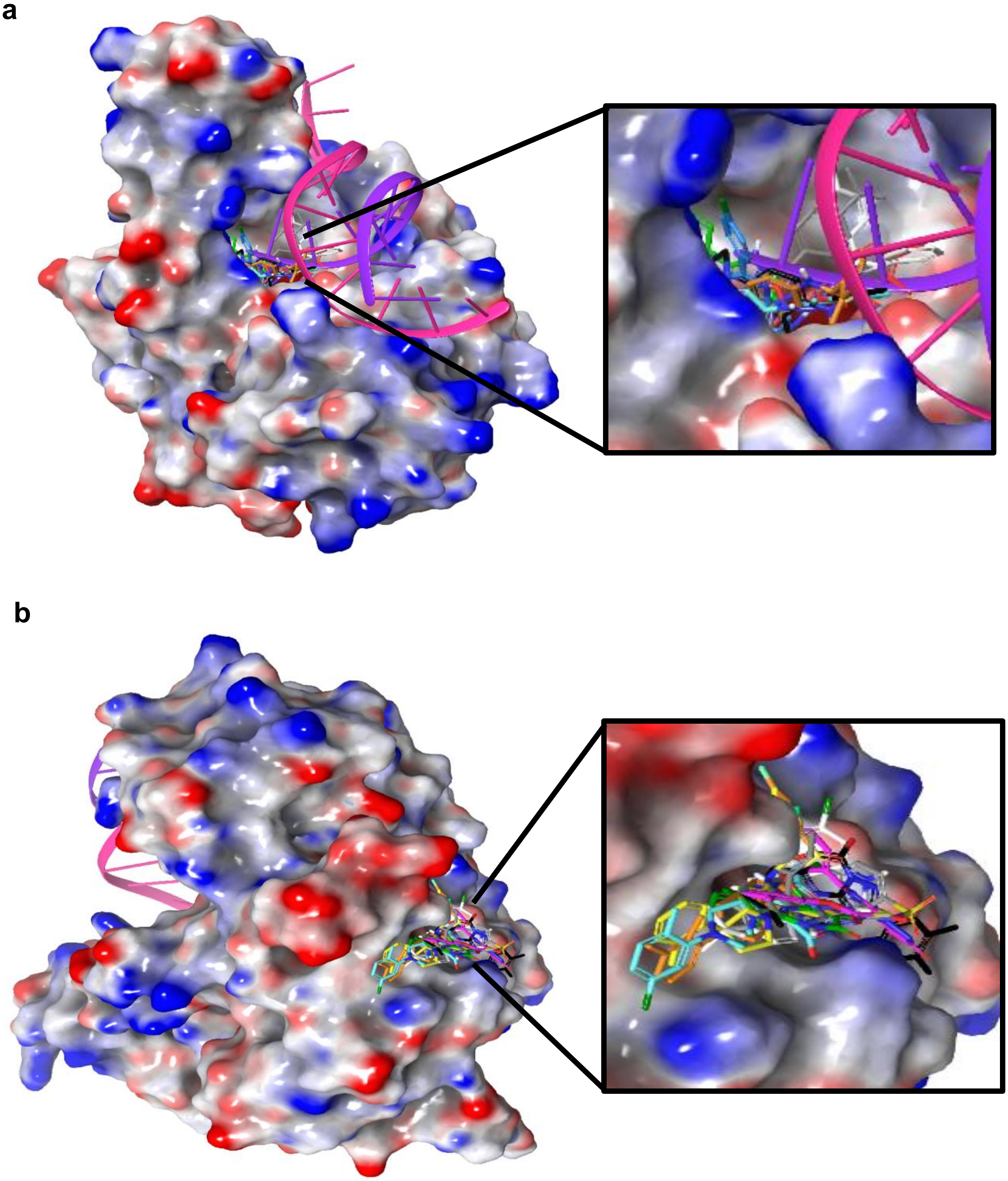

Figure S6. Inhibitor binding pockets of EXO1.
Three-dimensional structure of the **(a)** Site 1 binding pocket in the active site of EXO1, adjacent to the DNA substrate, and **(b)** Site 2 binding site on the anterior surface of EXO1. Blue color indicates areas of positive electron density, while red color shows negative electron density. DNA substrate strands are depicted in pink and purple. Black circles indicate the location of the Site 1 and Site 2 binding pockets. Druggable sites on the X-ray crystal structure of hEXO1 were identified via an in-house-developed Druggable Site Prediction by FDA-approved drugs (DSP) methodology, as previously described.[S1]

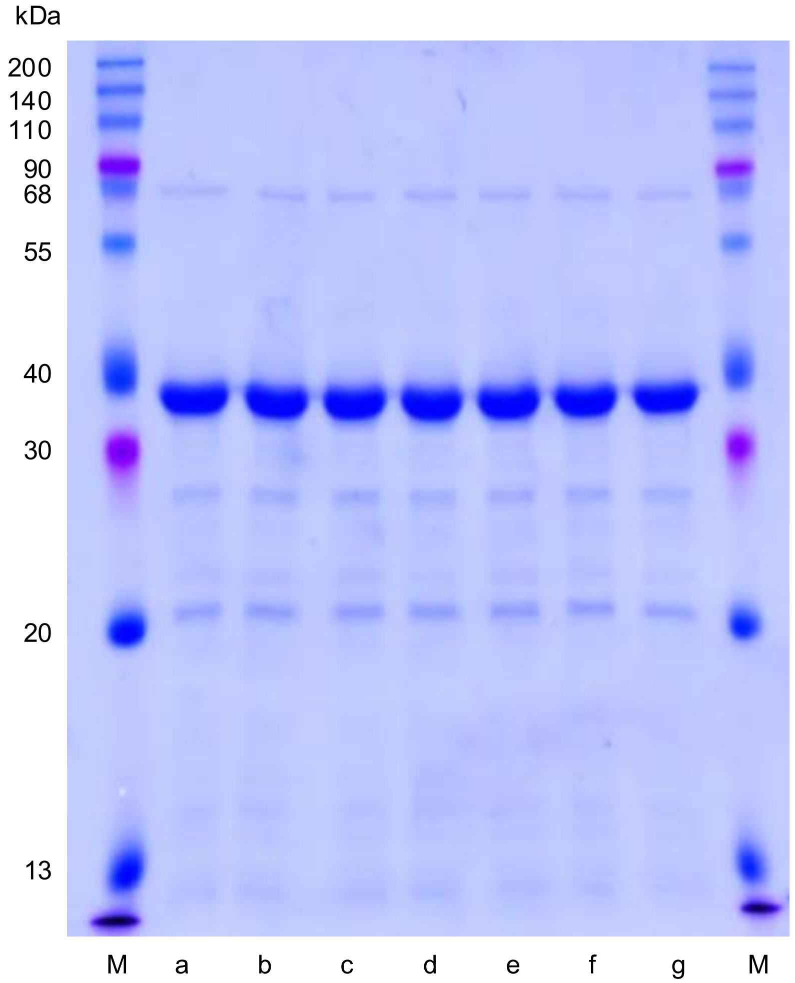

Figure S7. Purified EXO1 mutants.
SDS-PAGE gel showing the purity of purified recombinant 6xHis-tagged EXO1 N-terminal domain protein (residues 1-358; 38 kDa). **(a)** WT, **(b)** K85A, **(c)** E89A, **(d)** R92A, **(e)** I125A, **(f)** D225A, and **(g)** K292A-K294A recombinant proteins that were used in biochemical experiments. Lanes marked M contain the pre-stained protein marker; corresponding molecular weights are indicated to the left of the gel.

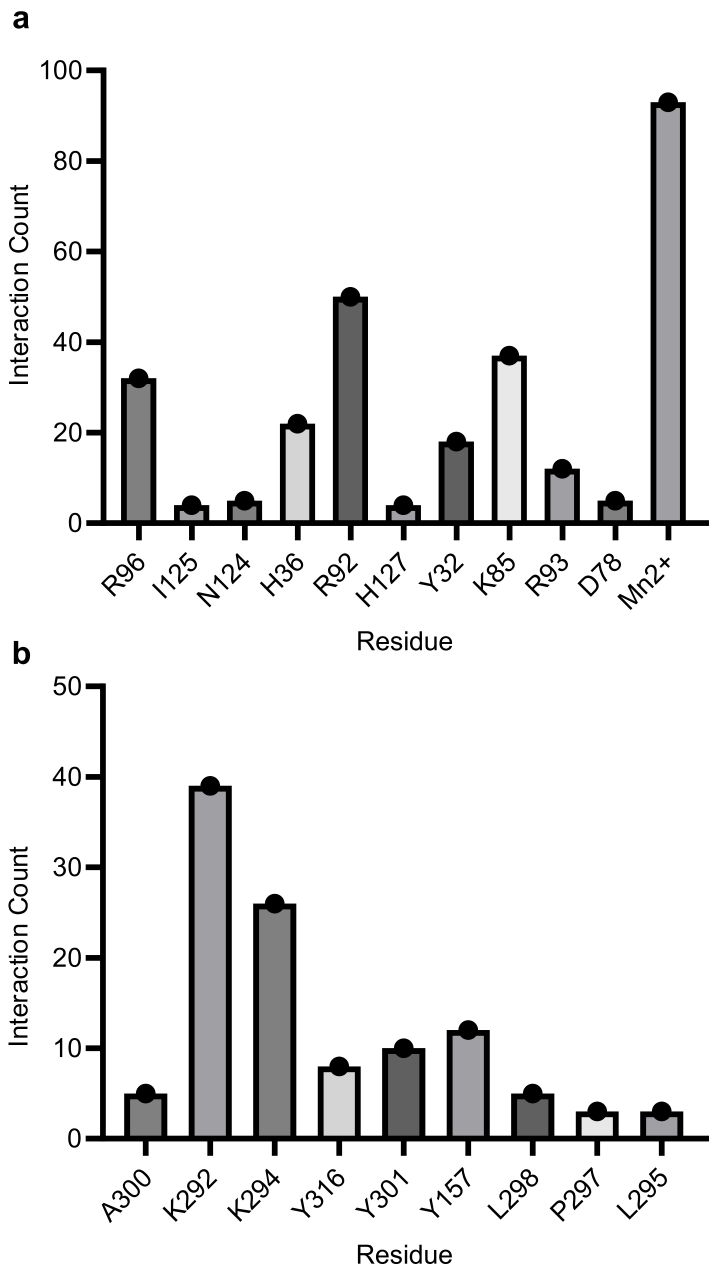

Figure S8. Binding of inhibitors within the Site 1 and Site 2 binding pockets. Indicates the total number of direct bonds between EXO1 and experimental compounds found within binding Site 1 **(a)** and Site 2 **(b)**, including hydrogen bonds, pi stacking, and salt bridges, observed between individual amino acids (or active site metal ions) and the seven inhibitor scaffolds; based on induced-fit docking analysis across various conformations of EXO1 (PDBs: 5UZV, 5V04, 5V05, 5V06, 5V07, 5V08, 5V09, 5V0A, 5V0B). Amino acid residues with the highest number of direct interactions with the inhibitor scaffolds were selected for mutagenesis, except for those known to be important for EXO1 nuclease function (i.e., H36 and Y32).[S2,S3]

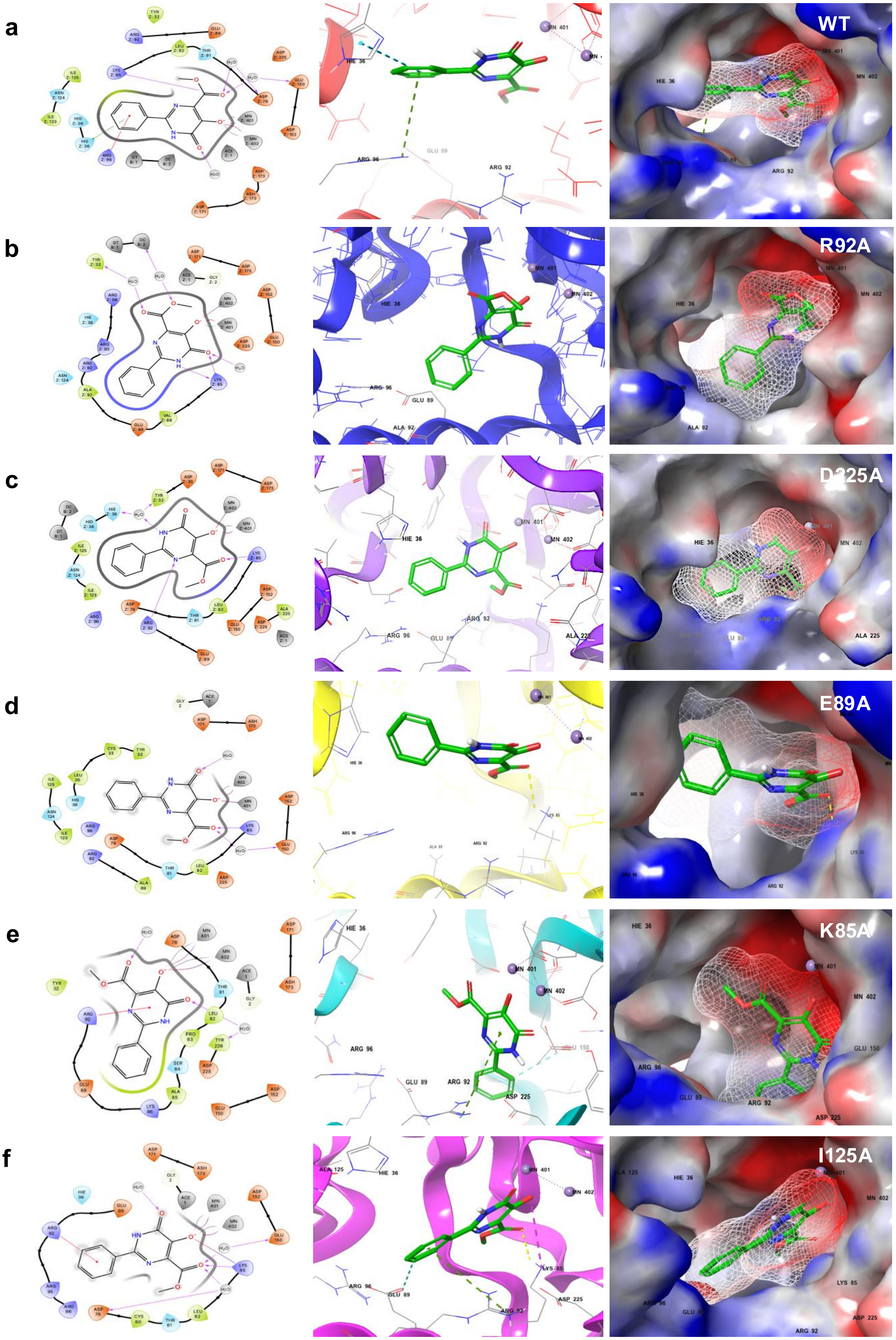
a
WT
b
R92A
c
D225A
d
E89A
e
K85A
f
I125A

Figure S9. Effect of Site 1 point mutations on C200 binding position.
Comparison of the chemical bonds and position of C200 within the EXO1 Site 1 binding pocket before and after point mutations. Left panel displays all residues within 5 Å of C200; pink lines = hydrogen bonds (H-bonds), green lines = pi-pi stacking interactions, red lines = pi-cation interactions, and red-blue gradient lines = salt bridges. Middle panel displays the proximity of C200 in relation to the two catalytic metal ions within the active site. Right panel displays the electron density of the protein surface surrounding C200; red color indicates a negative charge, and blue indicates a positive charge. Direct bonds between inhibitor C200 and each of the EXO1 variants include: **(a)** EXO1-WT: H-bonding between C200, Lys85, and several active site waters; salt bridging to Mn^{2+} ions; and pi-pi stacking with His36 and Arg96. **(b)** EXO1-R92A: H-bonding remained intact between C200, active site waters, Tyr32, and Lys 85, and salt bridging to Mn^{2+}. However, both pi interactions were lost. **(c)** EXO1-D225A: H-bonding between C200, Lys85, and active site waters, and salt bridging to Mn^{2+} remained. However, a new H-bond with Arg92 was gained, and both pi interactions were lost. **(d)** EXO1-E89A: H-bonding was sustained between C200, active site waters, and Lys 85; as was salt bridging to Mn^{2+}. Both pi interactions were lost. **(e)** EXO1-K85A: H-bonding with active site waters remained, as did salt bridging to Mn^{2+}. However, a new H-bond with Leu82, and pi stacking with Arg92 are observed. **(f)** EXO1-I125A: H-bonding between C200, Lys85, and active site waters was retained, as well as salt bridging with Mn^{2+}. A new pi interaction with Arg92 was observed.

a

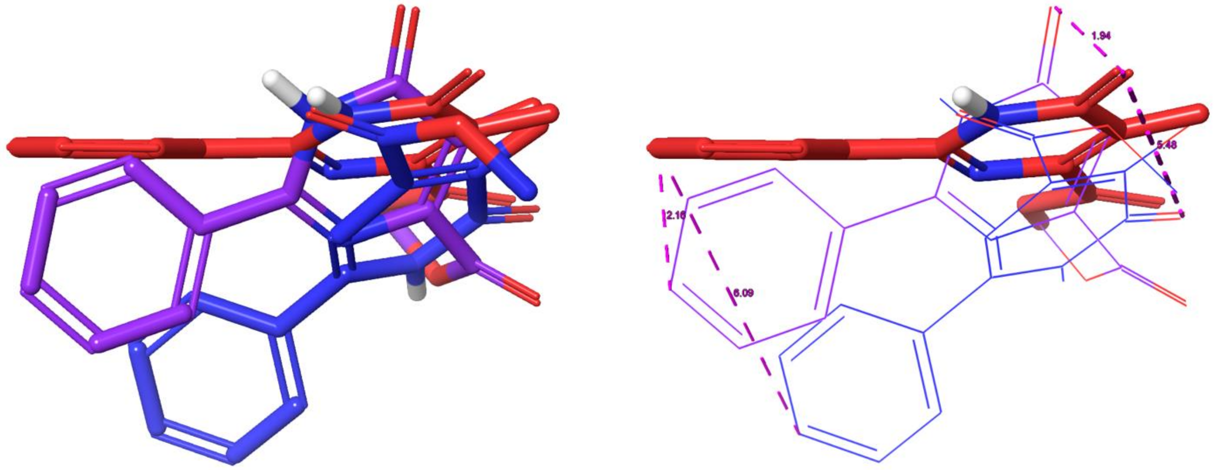

b

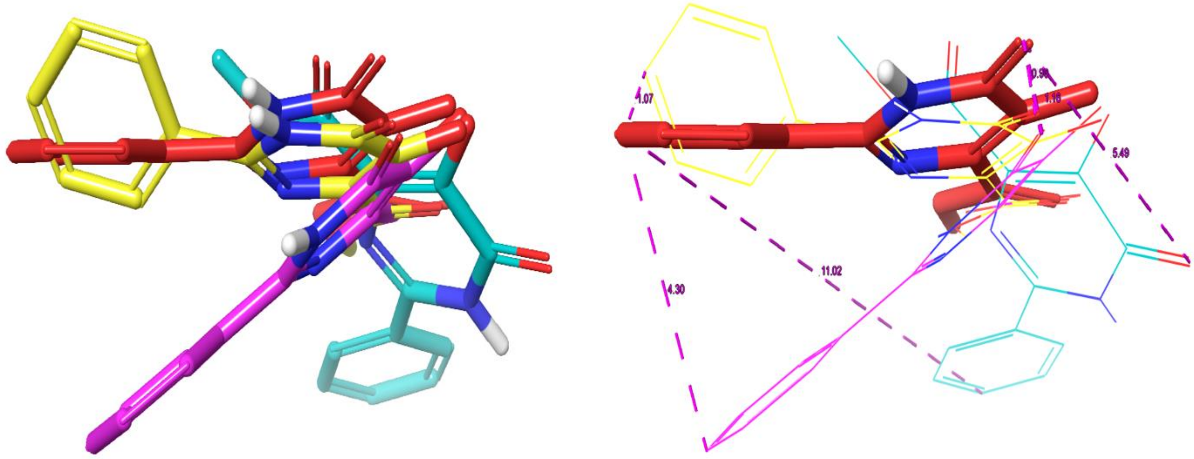

Figure S10. Positional comparison of C200 binding before and after Site 1 point mutations. Shown are how the binding position of C200 changes upon mutation of the Site 1 binding pocket. Color of the C200 compound corresponds to the ribbon color for each EXO variant as displayed in Figure S9. **(a)** EXO1-R92A and EXO1-D225A mutations resulted in a 6·09 Å (blue compound) and 2·16 Å (purple compound) deviation of C200's benzene ring from EXO1-WT (red compound). For both mutants, the benzene and pyrimidine rings of C200 are rotated > 45 °, significantly changing C200's position between 2 and 8 Å compared EXO1-WT. These positional changes corresponded with a loss of C200 inhibitory activity, measured via *in vitro* FRET-based assay. The distance between the binding positions of C200 in the R92A, D552A, and WT are indicated by pink dotted lines and the corresponding values. **(b)** Changes in C200 binding position within the Site 1 pocket of EXO1-WT (red compound), EXO1-E89A (yellow compound), EXO1-K85A (teal compound), and EXO1-I125A (magenta compound) are shown. E89A mutation slightly displaces the benzene ring of C200 by < 90°, resulting in a modest positional deviation of ~1·0 Å. In contrast, EXO1-K85A mutation resulted in a similar positioning of C200 to EXO1-WT, except with a ~90° clockwise rotation changed its overall position from 5·49 to 11·02 Å. Similarly, C200 binding against EXO1-I125A showed a ~45° downward shift of the benzene ring while the oxygen groups remain relatively unchanged, resulting in a lopsided positional change from EXO1-WT that ranged from 4·30Å at the benzene to 1·16Å at the opposing end. E89A, I125A, K85A mutants did not have a significant impact on C200 inhibitory activity *in vitro*, suggesting that positioning of the pyrimidine group of C200 in proximity to the metal ions is critical to EXO1 inhibition. Distance measurements of each mutant from EXO1-WT are indicated by pink dotted lines and values.

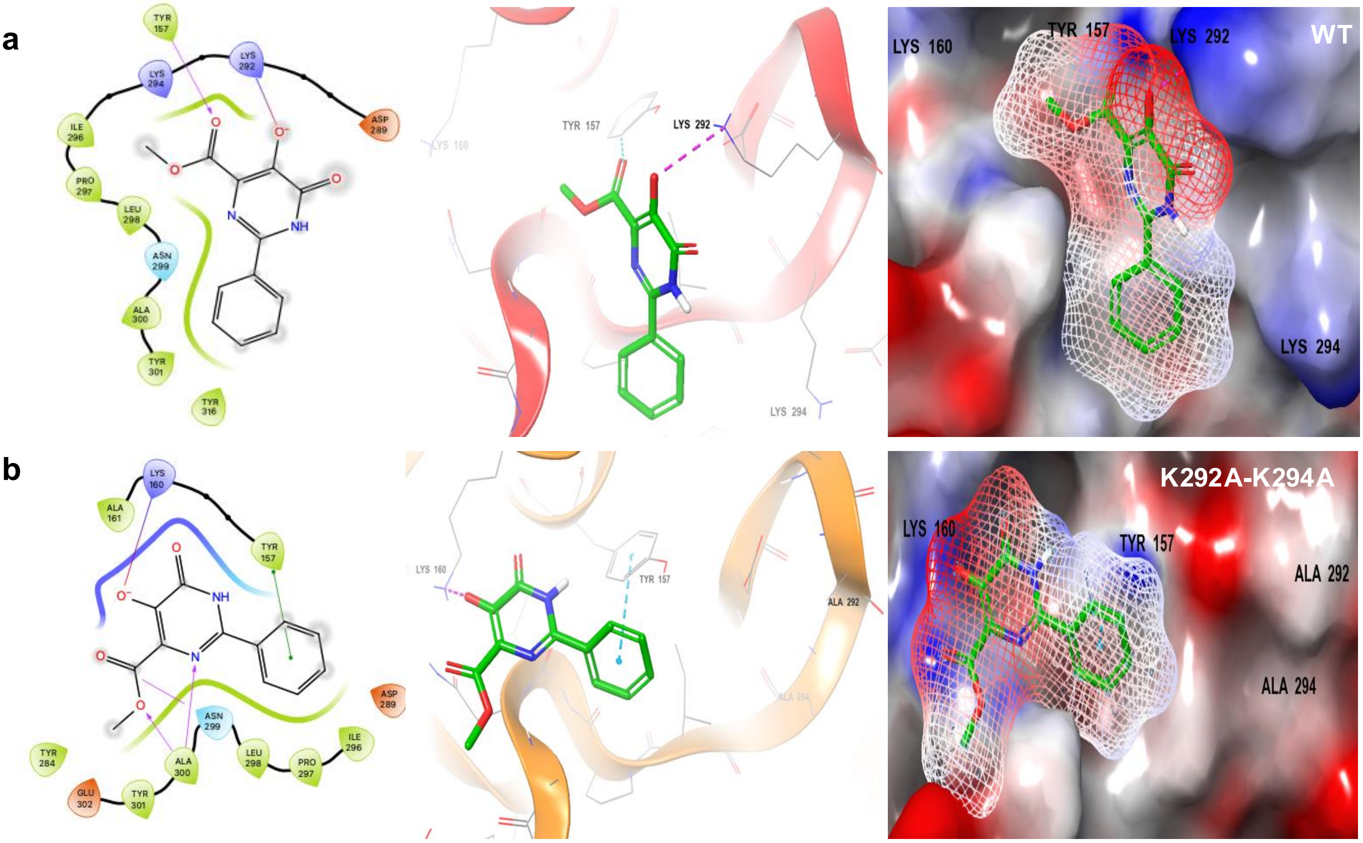

Figure S11. Effect of Site 2 point mutations on C200 binding position.
Comparison of the binding position and interactions of C200 within the EXO1 Site 2 binding pocket before and after point mutations. Left panel displays all residues within 5 Å of the compound; pink lines = H-bonds, green lines = pi-pi stacking interactions, red lines = pi-cation interactions, and red-blue gradient lines = salt bridges. Middle panel displays the proximity of C200 to the two catalytic metal ions within the EXO1 active site. Right panel displays the electron density of the protein surface surrounding C200; red color indicates negative charge and blue indicates positive charge. Chemical bonds between C200 and EXO1 Site 2 variants include: **(a)** EXO1-WT: H-bond is observed with Tyr157, and a salt bridge to Lys292. **(b)** EXO1-K292A-K294A: H-bond between Tyr157 is converted to a pi stacking interaction, and salt bridging has been transferred to Lys160. H-bonds with Asn299 and Ala300 are also gained. *In vitro*, C200 activity was lost upon K292A-K294A mutation, supporting Site 2 as an alternative binding site for EXO1 inhibitors.

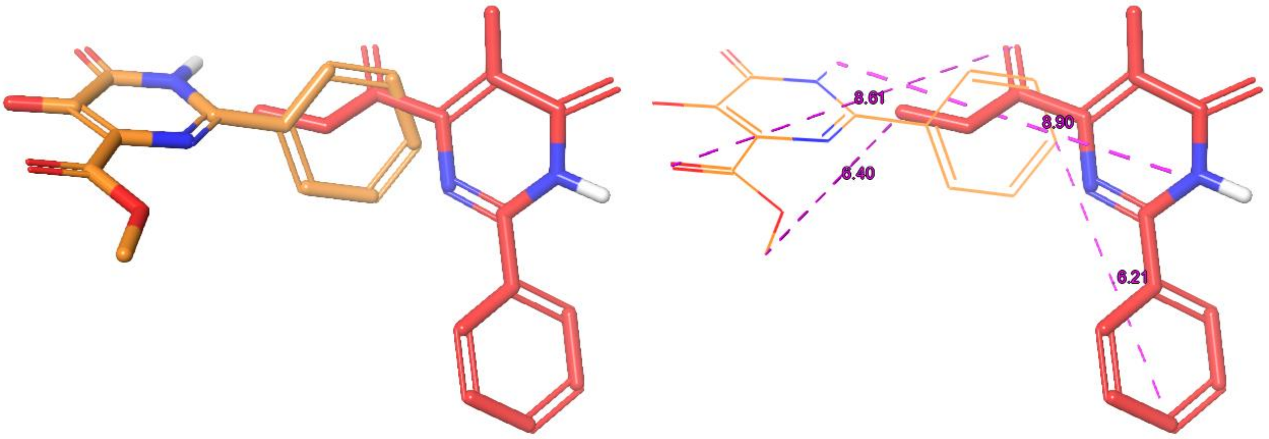

Figure S12. Positional comparison of C200 binding after Site 2 point mutations.
Shown are the changes in C200 binding position that occur when the Site 2 binding pocket is mutated. Color of the C200 compound corresponds to the ribbon color for each EXO variant as displayed in Figure S11. EXO1-K292A-K294A mutation caused a 6 to 8 Å deviation of C200 (orange compound) compared to EXO1-WT (red compound). This positional change corresponded with the loss of C200 inhibitory activity by FRET assay, suggesting that strong binding to Tyr157 via H-bonds may be essential for EXO1 inhibition via Site 2.

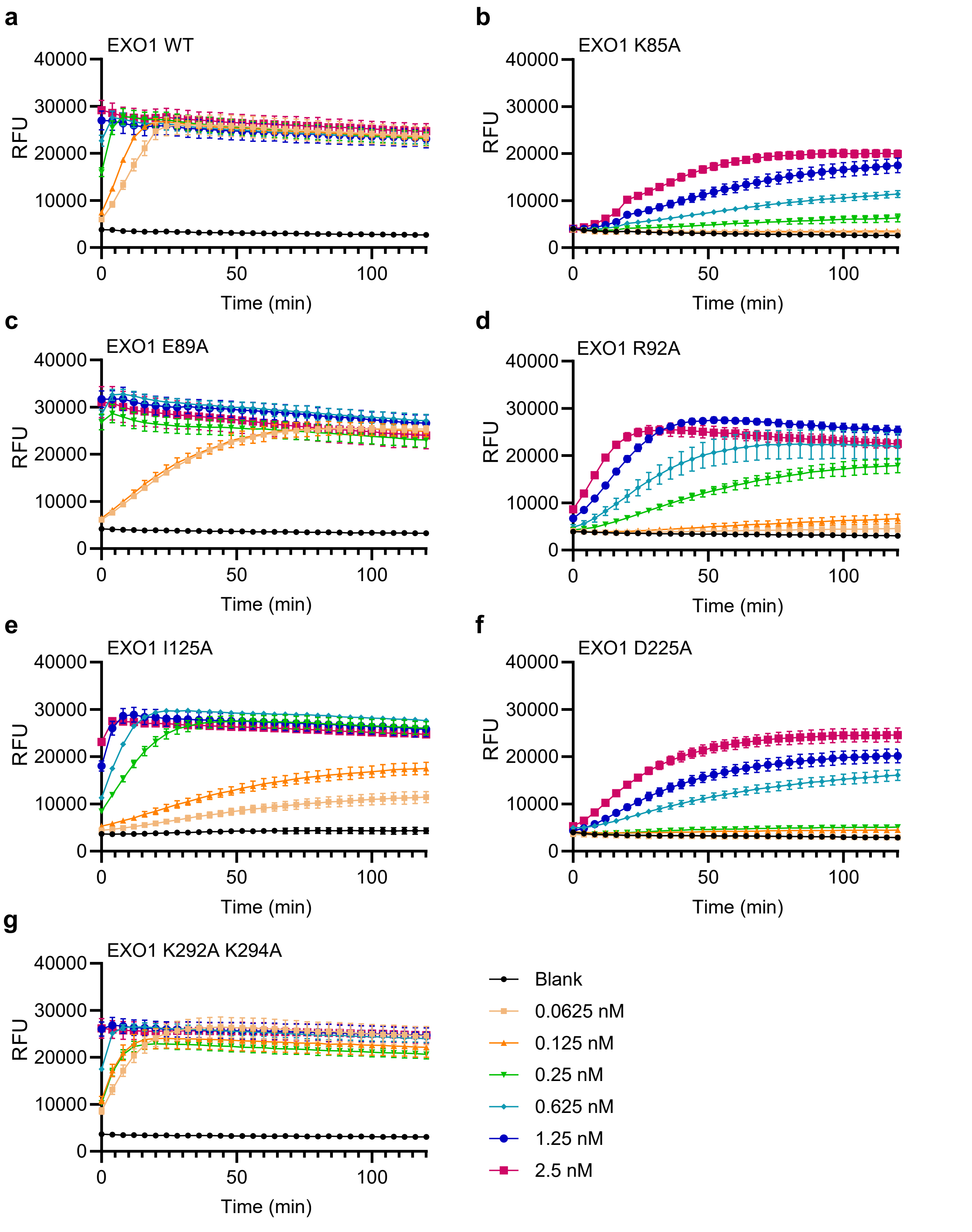

Figure S13. Effect of EXO1 point mutations on enzyme activity.
Comparison of the enzymatic activity of EXO1 before and after point mutations to Site 1 and Site 2 binding pockets. **(a)** EXO1-WT: full activity, max. signal = 31,125 RFU; **(b)** EXO1-K85A: moderate loss of activity, max. signal = 20,841 RFU; **(c)** EXO1-E89A: full activity, max. signal = 34,328 RFU; **(d)** EXO1-R92A: minimal loss of activity, max. signal = 27,015 RFU; **(e)** EXO1-I125A: minimal loss of activity, max. signal = 30,483 RFU; **(f)** EXO1-D225A: moderate loss of activity, max. signal = 25,543 RFU; **(g)** EXO1-K292A-K294A: minimal loss of activity, max. signal = 28,651 RFU.

Supplemental References

S1. Paiano J, Zolnerowich N, Wu W, et al. Role of 53BP1 in end protection and DNA synthesis at DNA breaks. *Genes Dev* 2021; **35**(19-20): 1356-67.
S2. Orans J, McSweeney EA, Iyer RR, et al. Structures of Human Exonuclease 1 DNA Complexes Suggest a Unified Mechanism for Nuclease Family. *Cell* 2011; **145**(2): 212-23.
S3. Shi Y, Hellinga HW, Beese LS. Interplay of catalysis, fidelity, threading, and processivity in the exo- and endonucleolytic reactions of human exonuclease I. *Proc Natl Acad Sci U S A* 2017; **114**(23): 6010-15.
